# Proteostatic modulation in brain aging without associated Alzheimer’s disease-and age-related neuropathological changes

**DOI:** 10.18632/aging.204698

**Published:** 2023-05-13

**Authors:** Pol Andrés-Benito, Ignacio Íñigo-Marco, Marta Brullas, Margarita Carmona, José Antonio del Rio, Joaquín Fernández-Irigoyen, Enrique Santamaría, Mónica Povedano, Isidro Ferrer

**Affiliations:** 1 Neurologic Diseases and Neurogenetics Group - Bellvitge Institute for Biomedical Research (IDIBE LL), L’Hospitalet de Llobregat, Barcelona 08907, Spain; 2 CIBERNED (Network Centre of Biomedical Research of Neurodegenerative Diseases), Institute of Health Carlos III, L’Hospitalet de Llobregat, Barcelona 08907, Spain; 3 Clinical Neuroproteomics Unit, Proteomics Platform, Proteored-ISCIII, Navarrabiomed, Complejo Hospitalario de Navarra (CHN), Universidad Pública de Navarra (UPNA), diSNA, Pamplona 31008, Spain; 4 Neuropathology Group, Institute of Biomedical Research, IDIBELL, L’Hospitalet de Llobregat, Barcelona 08907, Spain; 5 Molecular and Cellular Neurobiotechnology Group, Institute of Bioengineering of Catalonia (IBEC), Barcelona Institute for Science and Technology, Science Park Barcelona (PCB), Barcelona 08028, Spain; 6 Department of Cell Biology, Physiology and Immunology, Faculty of Biology, University of Barcelona, Barcelona 08007, Spain; 7 Department of Pathology and Experimental Therapeutics, University of Barcelona, L’Hospitalet de Llobregat, Barcelona 08907, Spain

**Keywords:** brain aging, cytoskeleton, membranes, synapsis, mitochondria, kinases, (phospho)proteomics, proteome

## Abstract

Aims: (Phospho)proteomics of old-aged subjects without cognitive or behavioral symptoms, and without AD-neuropathological changes and lacking any other neurodegenerative alteration will increase understanding about the physiological state of human brain aging without associate neurological deficits and neuropathological lesions.

Methods: (Phospho)proteomics using conventional label-free- and SWATH-MS (Sequential window acquisition of all theoretical fragment ion spectra mass spectrometry) has been assessed in the frontal cortex (FC) of individuals without NFTs, senile plaques (SPs) and age-related co-morbidities classified by age (years) in four groups; group 1 (young, 30–44); group 2 (middle-aged: MA, 45-52); group 3 (early-elderly, 64–70); and group 4 (late-elderly, 75–85).

Results: Protein levels and deregulated protein phosphorylation linked to similar biological terms/functions, but involving different individual proteins, are found in FC with age. The modified expression occurs in cytoskeleton proteins, membranes, synapses, vesicles, myelin, membrane transport and ion channels, DNA and RNA metabolism, ubiquitin-proteasome-system (UPS), kinases and phosphatases, fatty acid metabolism, and mitochondria. Dysregulated phosphoproteins are associated with the cytoskeleton, including microfilaments, actin-binding proteins, intermediate filaments of neurons and glial cells, and microtubules; membrane proteins, synapses, and dense core vesicles; kinases and phosphatases; proteins linked to DNA and RNA; members of the UPS; GTPase regulation; inflammation; and lipid metabolism. Noteworthy, protein levels of large clusters of hierarchically-related protein expression levels are stable until 70. However, protein levels of components of cell membranes, vesicles and synapses, RNA modulation, and cellular structures (including tau and tubulin filaments) are markedly altered from the age of 75. Similarly, marked modifications occur in the larger phosphoprotein clusters involving cytoskeleton and neuronal structures, membrane stabilization, and kinase regulation in the late elderly.

Conclusions: Present findings may increase understanding of human brain proteostasis modifications in the elderly in the subpopulation of individuals not having AD neuropathological change and any other neurodegenerative change in any telencephalon region.

## INTRODUCTION

Brain aging is a progressive detrimental process manifested by specific structural, molecular, and functional changes with a variable individual pace. Brain aging is complex because it affects different neuronal types, connections, astroglia, microglia and oligodendroglia, blood vessels, and other intrinsic and systemic cell populations. Moreover, aging does not work according to simple cause-effect logic. Outcomes are not directly caused by a single driver but emerge from multiple, non-related, and combined events following non-cartesian, non-linear logic. Alterations of nucleotides, nucleic acids, proteins, lipids, carbohydrates, and metabolites occur at individual cellular levels. Such alterations are part of the molecular substrates which result in impaired brain function in the aged brain and human elderly. Human brain aging is also linked with numerous neurodegenerative diseases such as dementia of Alzheimer’s [1, 2].

The present study is focused on protein changes in the aged human frontal cortex. Previous whole proteomics studies have identified modifications in the expression levels of proteins during human brain aging [3–18]. Relevant conclusions can be summarized along these lines: (i) there is progressive deregulation of protein expression in the elderly; (ii) there are marked individual variations; (iii) there are regional differences within the same age range; and (iv) multiple structures and metabolic pathways are targeted by altered protein expression levels.

Most studies in humans are performed using brain samples with age-dependent progressive accumulation of Alzheimer’s disease-related pathology (or AD neuropathological changes: ADNC), named neurofibrillary tangles (NFTs) and senile plaques (SPs). This situation is not strange as about 85% of the human population has NFTs at least in the deep temporal cortex (corresponding to stages I, II or IIII) without cognitive impairment at 65; about 98% have more extensive NFT pathology and 30% dementia at 80. About 30% has SPs at the age of 65, whereas SPs are found in around 60% of individuals over 80 [1, 19–25]. As a result, most if not all studies of the proteome in human brain aging are carried out in samples of individuals having variable degree of ADNC. Those without cognitive impairment labelled as normal for age (but usually having NFT pathology at Braak stages I–III) were compared with individuals with mild cognitive impairment or dementia of Alzheimer’s type having large numbers of NFTs and SPs.

Phosphorylation is one of the most common and essential mechanisms of protein regulation throughout activation or inhibition of protein function and interaction of recruited proteins [26–31]. Several studies have identified deregulated protein phosphorylation in sAD [10, 32–39], even at first stages (stages I-II) of NFT pathology in the frontal cortex in which no NFTs and SPs are present at these stages [38]. Differentially regulated phosphoproteins are components of cell membranes and membrane signaling, cytoskeleton, synapses including neurotransmitter receptors, serine-threonine kinases, proteins involved in energy metabolism, and RNA processing and splicing. Deregulated protein phosphorylation is more pronounced at stages III and IV; it is maintained at advanced stages of AD [38]. Altered brain protein phosphorylation also occurs in human tauopathies [40, 41]; it has also been described in transgenic mouse models of cerebral β-amyloidosis, and tauopathy [42–47]. To our knowledge, whole phosphoproteomes centered on the human brain aging without AD pathology are unavailable.

The purpose of the present study was to obtain proteomics and phosphoprotemics data of the frontal cortex (FC) at different age stages, from 30 to 85 years, in individuals without NFTs and SPs pathology in any brain region of the telencephalon. In addition, these subjects had not concomitant age-related pathologies such as TDP-43 proteinopathy (LATE), α-synucleinopathy, other tauopathies such as argyrophilic grain disease, frontotemporal lobar degeneration (FTLD), hippocampal sclerosis, and vascular diseases. Conventional label-free- and SWATH-MS (Sequential window acquisition of all theoretical fragment ion spectra mass spectrometry) were used to assess the (phospho)proteomes across age groups in the FC. A subgroup of proteins was validated using immunohistochemistry and or western blotting. The limited number of samples (*n* = 4) in every group is due to the extreme rarity of finding elderly persons without ADNC and any other neuropathological lesion.

## RESULTS

To quantify age-dependent fluctuations in the frontal cortex (FC) on a proteome-wide scale, we performed an integrative analysis of the proteome and phosphoproteome across age stages classified by age (years) in four groups: group 1 (young): 30–44, group 2 (middle-aged: MA): 45–52, group 3 (early-elderly): 64–70, and group 4 (late-elderly): 75–85. Although the first group was composed of men only, the distribution of sexes was similar in the other groups. As shown in principal component and dendrogram analyses, the quantified proteomes and phosphoproteomes are not biased in any group, suggesting that there is not a clear effect of the sex variable (Supplementary Figure 1).

### Proteome and phosphoproteome dynamics in the FC: functional enrichment analysis

**Figure 1:**
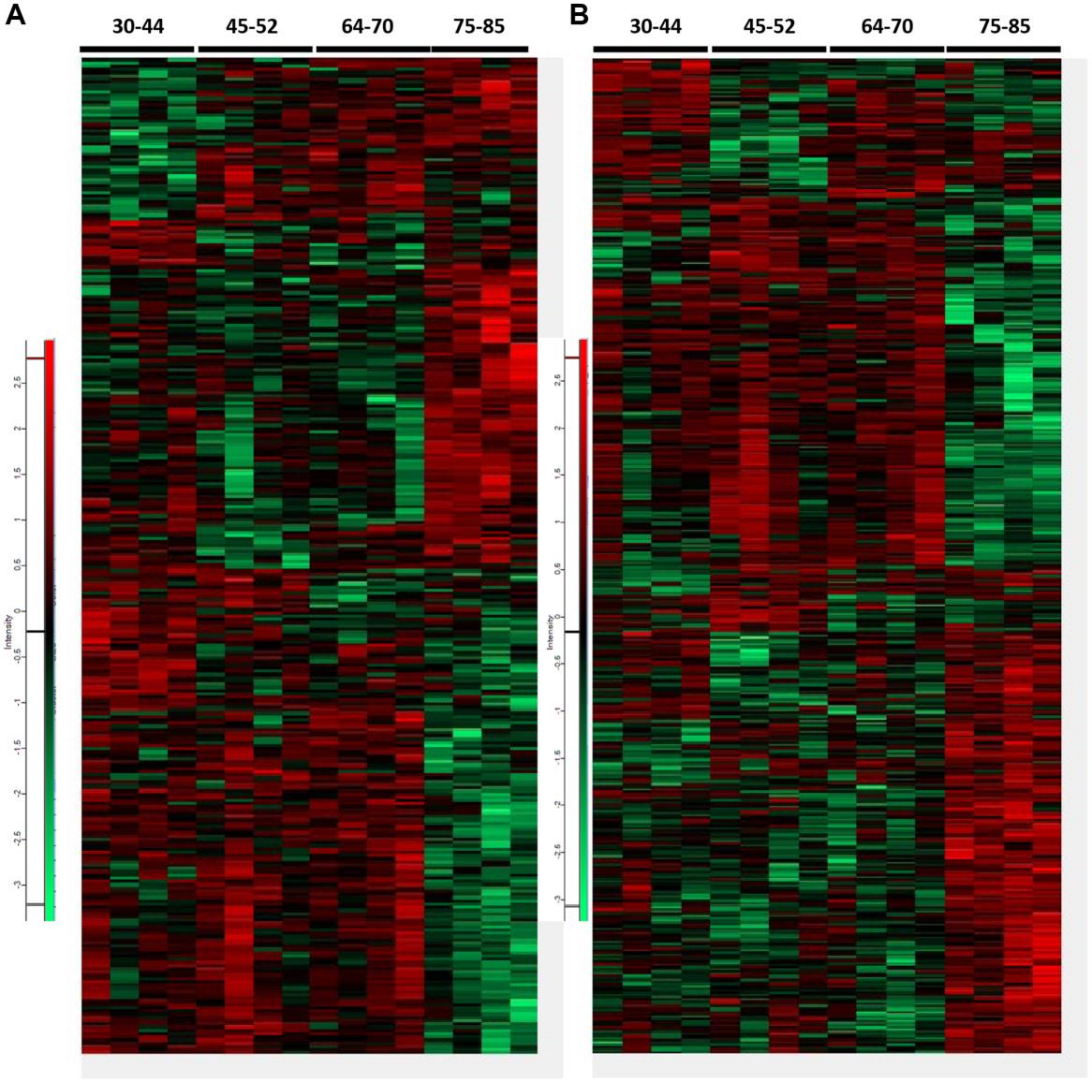
Heatmaps representing differential proteins and phosphopeptides at proteome (**A**) and phosphoproteome (**B**), respectively, across all age groups. A total of 308 differentially expressed proteins and 465 differential phosphopeptides were identified when comparing all groups (ANOVA significant, *p* < 0.05). Among them, 280 proteins and 278 phosphopeptides were common to all groups in proteome and phosphoproteome analysis (*p* < 0.05). Only 28 proteins with altered expression levels also showed deregulated phosphorylation. Increased levels are indicated in the red spectrum, whereas decreased levels in the green spectrum.

Heatmaps of proteomes and phosphoproteomes showed marked differences in the FC across age groups. A total of 2,830 proteins and 6,722 phosphopeptides were quantified. 308 differential expressed proteins and 465 differential phosphopeptides (306 proteins) were identified when comparing all age groups using the ANOVA one-way test (*p* < 0.05; Fold Change > 30%) (Figure 1). Two hundred eighty differentially expressed proteins and 278 phosphopeptides were familiar to the four groups (*p* < 0.05). Protein levels and the direction of hyper- or hypo-phosphorylation were variable in every age group. Some phosphoproteins showed more than one phosphorylation site; the direction of phosphorylation varied in every phosphosite. Only 28 deregulated phosphoproteins also showed altered total expression levels. These numbers indicate that only a tiny percentage of deregulated phosphoproteins might correspond to abnormal expression levels of the corresponding protein.

**Figure 2:**
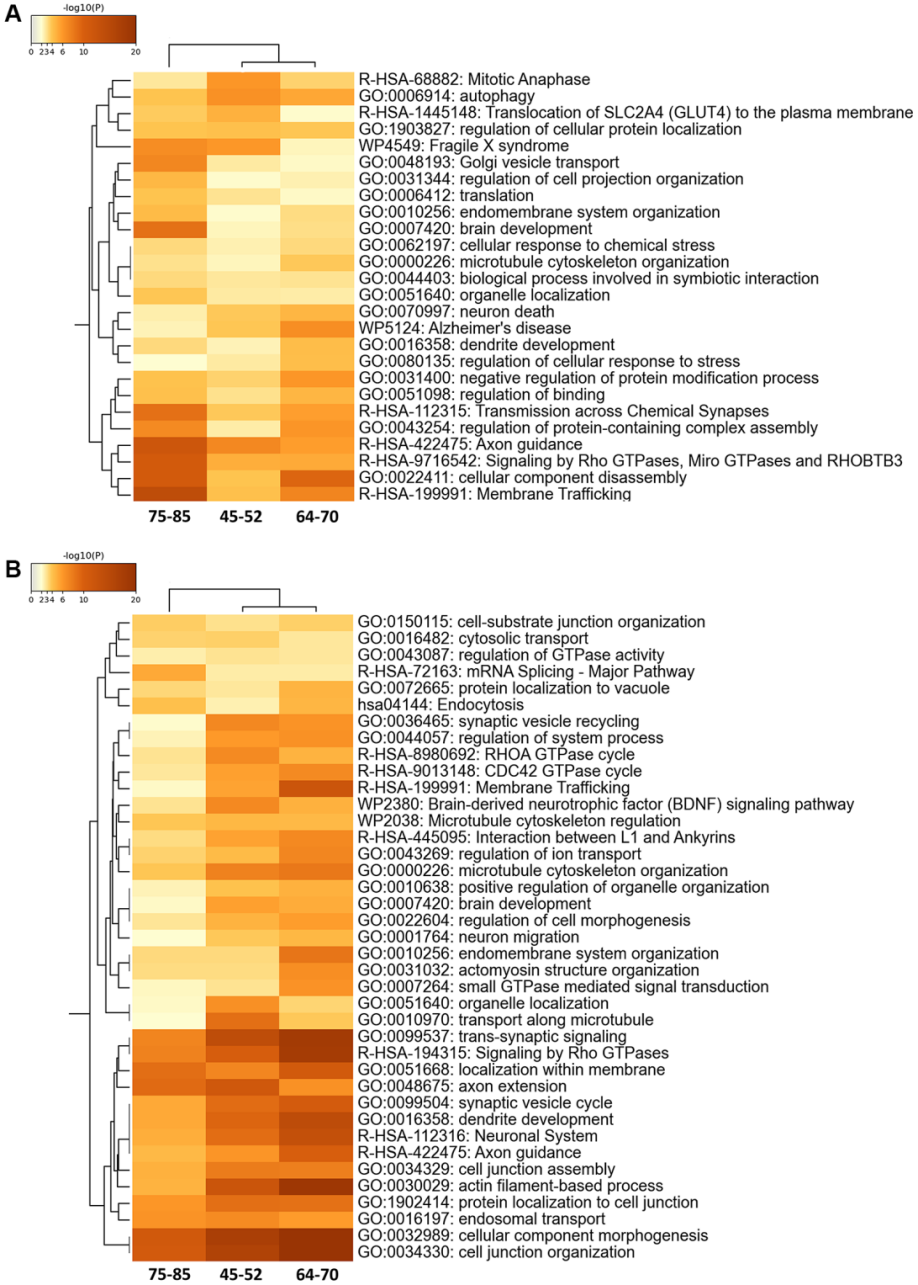
Enriched ontology clusters in the 30% FC differential proteomes (**A**) and phosphoproteomes (**B**) during aging using Metascape. After identification of all statistically enriched terms, cumulative hypergeometric *p*-values and enrichment factors were calculated and used for filtering. The remaining significant terms were then hierarchically clustered into a tree based on Kappa-statistical similarities among their gene memberships. Then, a 0.3 kappa score was applied as the threshold to cast the tree into term clusters. The term with the best *p*-value within each cluster was selected as its representative term and displayed in a dendrogram.

The proteostatic modulation across age stages was assessed by merging and functionally analyzing differential proteomic and phosphoproteomic datasets according to specific biological functions. We evaluated age-related functional alterations using group 1 (young) as a control group. Protein alterations overlapped across age stages and were accompanied by a considerable overlap in enriched GO terms. Main altered terms/functions were associated with membrane trafficking, microtubule cytoskeleton organization, axon guidance, and GTPase alterations related to vesicles and endomembrane processes (Figure 2A).

Phosphoprotein alterations also overlapped across age stages and were accompanied by a considerable overlap in enriched GO terms. Yet, the phosphoproteome showed more functional alterations associated with GO terms than the proteome. Alterations also included those linked to cytostructural functions such as cell-substrate junction organization, interaction between L1 and ankyrins, microtubule cytoskeleton regulation, cell morphogenesis, actomyosin structure organization, axon extension, dendrite development, cell junction assembly, actin filament-based processes, protein localization to cell junction, and cell junction organization (Figure 2B).

**Figure 3:**
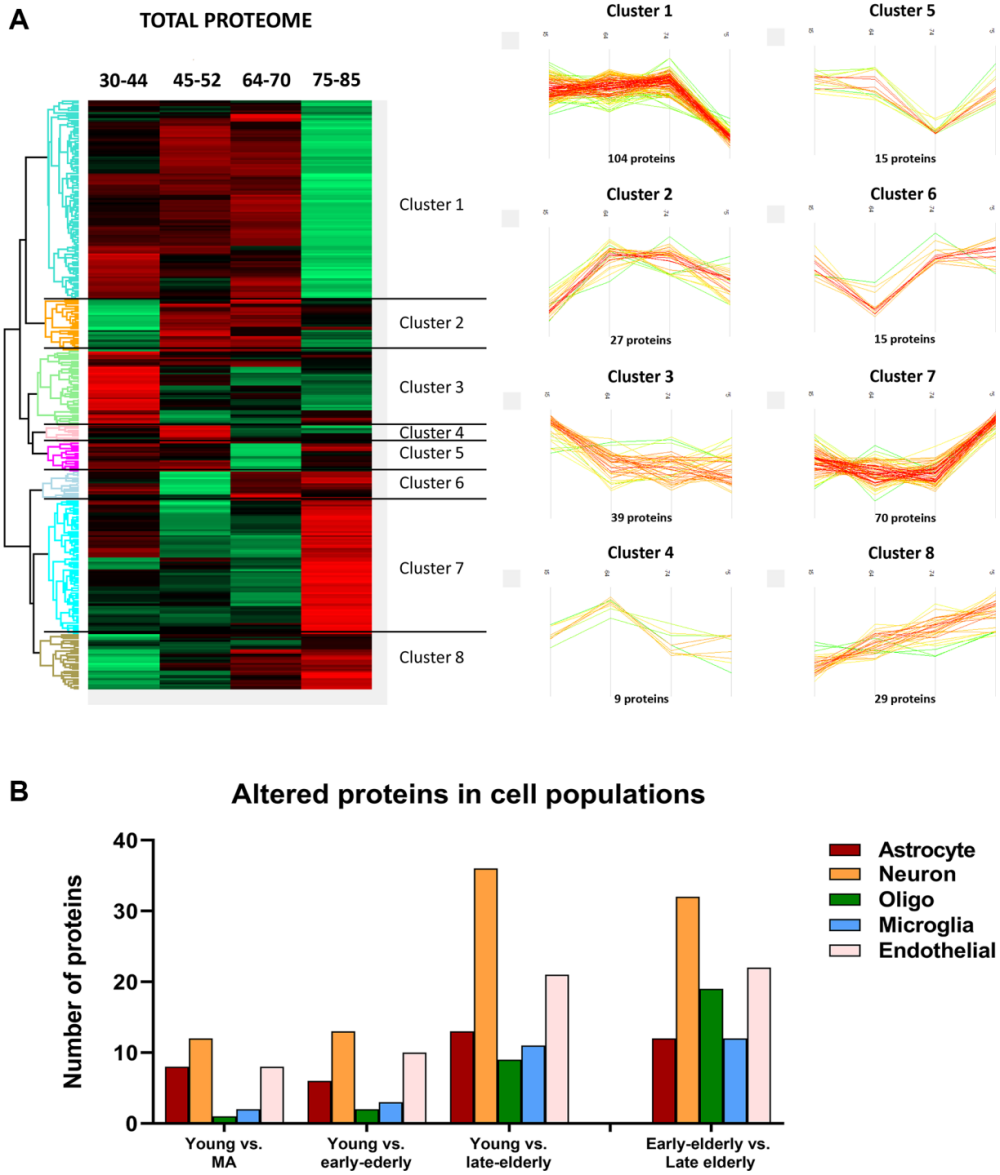
(**A**) Differentially expressed proteins across age. (Left) Heatmap representing the differential expressed proteins across the four age groups: group 1 (young): 30–44y; group 2 (middle-aged: MA): 45–52y; group 3 (early-elderly): 64–70y; and group 4 (late-elderly): 75–85y. Each line corresponds to a protein, in which the Z-score is represented as a numerical measurement that describes the relationship between averaged protein intensity values in a specific condition and the mean intensity for each protein across all experimental conditions. The Z-score (considered a measurement in terms of standard deviations from the mean) may be positive (scoring above the mean; represented in red) or negative (scoring below the mean; represented in green). (Right) Profile-plots representing protein clusters with similar expression trajectories across age. The most representative clusters show protein groups specifically down-regulated (Cluster 1) or up-regulated (Cluster 7) in the late-elderly group. (**B**) The graphical representation illustrates the cellular type assignment of proteins based on available RNA-seq databases. The major changes are observed in proteins associated with neurons.

**Figure 4:**
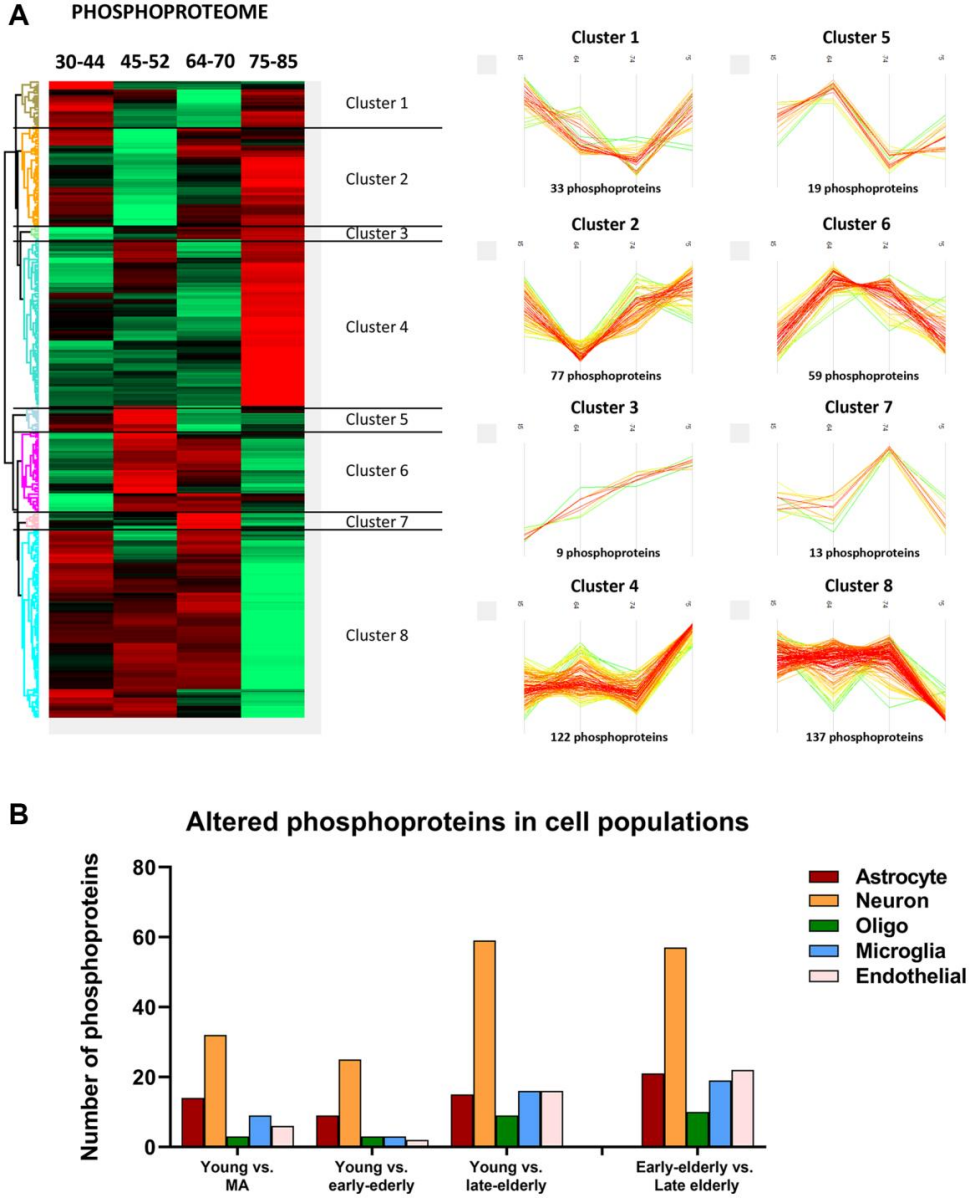
(**A**) Monitoring of differentially expressed phosphoproteins across age. (Left) Heatmap representing the differential expressed phosphorylated proteins across the four age groups: group 1 (young): 30–44y; group 2 (middle-aged: MA): 45–52y; group 3 (early-elderly): 64–70y; and group 4 (late-elderly): 75–85y. As indicated in Figure 2, each line corresponds to a phosphoprotein in which the Z-score (a measurement in terms of standard deviations from the mean) is evaluated. Positive and negative Z-scoring is represented in red and green respectively. (Right) Profile-plots representing phosphoprotein clusters with similar expression trajectories across age. Cluster 4 and cluster 8 indicate protein subsets that are specifically modulated in the late-elderly group. (**B**) The graphical representation illustrates the cellular type assignment of phosphoproteins based on available RNA-seq databases. The major changes are observed in proteins associated with neurons.

Then, deregulated proteins and phosphoproteins were categorized into eight clusters based on their age-dependent expression similarity. Interestingly, protein and phosphoprotein levels of the larger hierarchical clusters were stable until the age of 70 years. After this age, the late-elderly group showed decreased or increased expression of the two major protein clusters, 1 and 7, respectively (Figure 3A). Similarly, major phosphorylation modifications occurred in the late-elderly group in clusters 4 and 8 (Figure 4A). Proteins and phosphoproteins composing every cluster are detailed in the Supplementary Table 1.

Additionally, to learn about cell-type-specific molecular signatures, we conducted a multi-comparative analysis comparing our protein data with available cell-type databases of RNA-seq. This procedure allowed the categorization of altered (phospho)proteins as neuronal, astroglial, oligodendroglial, microglial, and endothelial. The main proteomic alterations were related to neuronal populations in all groups. However, these changes were more pronounced with age (Figure 3B). A similar pattern was observed regarding phosphoproteomics data: the higher number of altered phosphoproteins was related to neuronal populations, and major changes were seen in groups 3 and 4 compared with group 1 (Figure 4B).

**Figure 5:**
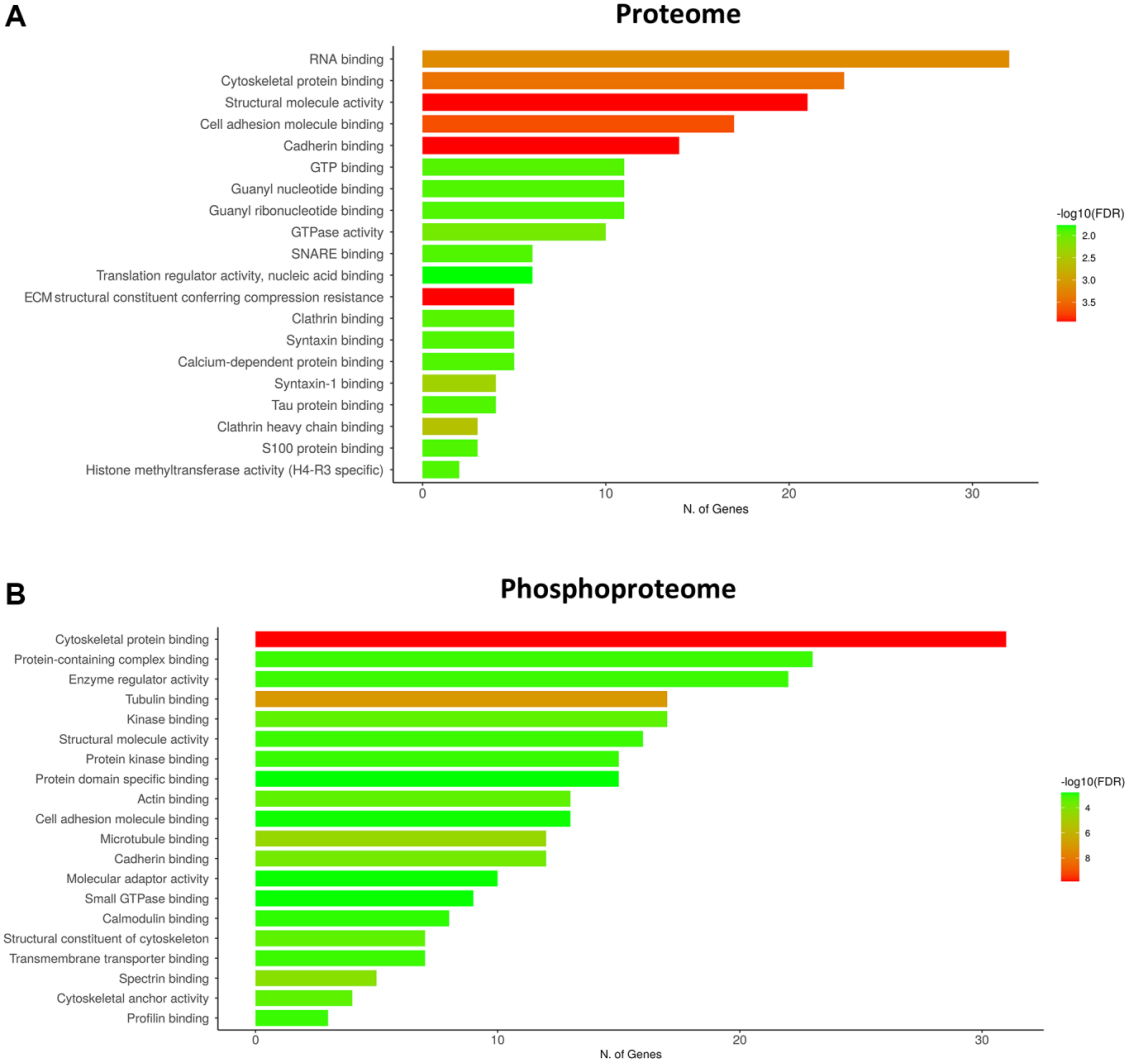
**GO terms of the molecular functions of the main deregulated clusters in the late-elderly group (group 4).** (**A**) Molecular functions of GO-enrichment analysis reveals that the main altered proteins in clusters 1 and 7 in late-elderly are related to cell membranes functions, vesicle and synaptic functions, RNA binding, and structural components. (**B**) Deregulated phosphoproteins in clusters 4 and 8 in the late-elderly group are principally connected with cytoskeleton composition and regulation, kinase regulation and membrane stabilization. Although GO is referred to gene ontology, we have chosen the term number of proteins instead to avoid confusions.

As observed in Figures 3 and 4, the increase or decrease in protein levels and protein phosphorylation depends on the age-stage in each case, exhibiting a mixture of patterns. However, a significant number of proteins show a consistent pattern that changed in the late-elderly group. These patterns encompass over 50% of the proteins and phosphoproteins. Functional analysis was performed on 174 of 308 proteins and 259 of 469 phosphoproteins that increased or decreased in the late-elderly group. No attempt was made to differentiate between increased or decreased protein expression levels, and between hypo- and hyperphosphorylation because the functional implications of hyper-and hypo-phosphorylation at specific sites are not known for the majority of phosphoproteins. Yet, molecular functions of GO-enrichment analysis revealed that the main altered proteins in clusters 1 and 7 in group 4 were linked to cell membranes functions, vesicle and synaptic functions, RNA binding, and structural components (Figure 5A). GO-molecular functions of deregulated phosphoproteins in clusters 4 and 8 in the group 4 were mainly connected with the cytoskeleton composition and regulation, kinase regulation and membrane stabilization (Figure 5B).

### Individualized analysis of proteins with modified levels and phosphorylated proteins expressed in the FC at different aging stages

Deregulated proteins were individually categorized according to their localization and function in the CNS. Cytoskeletal (*n* = 47) and membrane proteins (*n* = 77), including proteins of the synapses and vesicles, myelin proteins, and proteins linked to membrane transport and ion channels, accounted for 124 differentially-expressed proteins. This classification was instrumental as some membrane proteins also participate in the structure of the synapses, and several synaptic proteins are plasma membrane proteins. Other deregulated proteins were related to DNA and RNA metabolism (*n* = 48), ubiquitin-proteasome-system (UPS) (*n* = 17), and kinases and phosphatases (*n* = 31). The remaining 83 proteins participated in other functions such as GTPases, fatty acid metabolism, and mitochondria. Their symbols, full names, and primary functions are summarized in Supplementary Tables 2–7.

The largest group of deregulated phosphoproteins were associated with the cytoskeleton and integral membrane proteins (*n* = 99), including in the first categorization, microfilaments, actin-binding proteins, intermediate filaments of neurons and glial cells, and microtubules. A second group was formed by phosphoproteins of the membranes, synapses, and dense core vesicles (*n* = 74). Other deregulated phosphoproteins were categorized as kinases and phosphatases (*n* = 36), proteins linked to DNA and RNA (*n* = 44), and members of the UPS (*n* = 16). The remaining 36 deregulated phosphoproteins involved GTPase regulation, inflammation, and lipid metabolism. Their symbols, full names, and primary functions are shown in Supplementary Tables 8–13.

### Proteomic and phosphoproteomic data validation using Western blotting and immunohistochemistry

**Figure 6:**
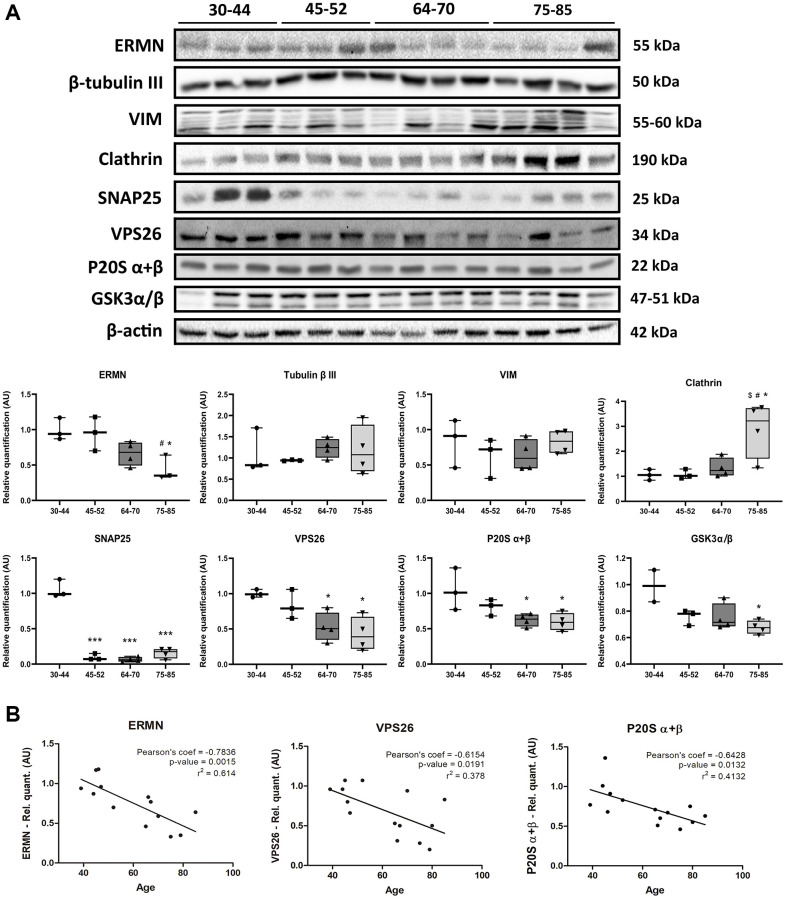
(**A**) Gel electrophoresis and western blotting to ermin (ERMN), β-tubulin III, vimentin (VIM), clathrin (heavy chain), SNAP25, VPS26, P20S α + β, and GSK3α/β in the FC area 8 across age; group 1 (young): 30–44y, group 2 (middle-aged: MA): 45–52y; group 3 (early-elderly): 64–70y; and group 4 (late-elderly): 75–85y. Significantly decreased expression levels of ERMN are found in group 4 compared with group 1 and group 2 (*p* < 0.05). Clathrin expression is significantly increased in group 4 compared with groups 1, 2, and 3 (*p* < 0.01). In contrast, there is a significant reduction of SNAP25 levels in groups 2, 3, and 4 compared with group 1 (*p* < 0.001), and VPS26 in groups 3 and 4 compared with group 1 (*p* < 0.05). P20S α + β levels are reduced in groups 3 and 4 compared with group 1 (*p* < 0.05). Similarly, reduced levels in GSK3α/β are observed in aging, reaching significant differences between group 1 and group 4 (*p* < 0.05). (**B**) Decreased levels of ERMN, VPS26 and P20S α + β significantly correlate with age. Pearson’s correlation significance level set at *p* < 0.05.

We validated changes in structural components of the cytoskeleton using gel electrophoresis and western blotting with antibodies anti-ermin (ERMN), β-tubulin III, and vimentin (VIM). Protein levels were assessed by densitometry, and a one-way ANOVA was performed to compare the effect of aging between groups on protein levels. One-way ANOVA revealed that there was a statistically significant difference in ERMN between at least two groups (F_(3,9)_ = 5.912, *p* = 0.0164). Tukey’s HSD Test for multiple comparisons found that the mean value of ERMN was significantly different between late-elderly group (group 4) when compared with groups 1 and 2 (*p* < 0.05). β-tubulin III did not change with age (F_(3,10)_ = 0.3102, *p* = 0.8176). Vimentin expression was increased in the three cases aged 47–85 and two cases aged 60–70. However, statistical analysis of all the samples did not show significant differences among the four groups (F_(3,10)_ = 0.7277, *p* = 0.5584 (Figure 6A).

Levels of proteins related to vesicular components were also evaluated. One-way ANOVA revealed a significant difference in clathrin levels between at least two groups (F_(3,10)_ = 6.566, *p* = 0.0099). Post-hoc Tukey’s test indicated that clathrin levels were significantly increased in the late-elderly group compared with groups 1, 2, and 3 (*p* < 0.05 for each group) (Figure 6A). One-way ANOVA test also showed a significant difference in SNAP25 and VPS26 levels between at least two groups (F_(3,10)_ = 127.9, *p* < 0.0001) and (F_(3,10)_ = 6.166, *p* = 0.0121), respectively). Tukey’s HSD Test for multiple comparisons found that the mean value of SNAP25 levels was significantly decreased groups 2, 3, and 4 (*p* < 0.001 for each group compared young group (group 1). Similarly, Tukey’s HSD Test for multiple comparisons found that VPS26 levels were significantly reduced comparing group 1 with group 3 (*p* < 0.05) and group 4 (*p* < 0.05) (Figure 6A).

Furthermore, the components of the proteasome system were also studied using western blotting. One-way ANOVA revealed no altered levels in the P19S proteasome protein (F_(3,10)_ = 1.167, *p* = 0,3703), and LMP2 (F_(3,10)_ = 1.193, *p* = 0,3614) and LMP7 (F_(3,10)_ = 0.3329, *p* = 0.8019) inflammatory-modulated subunits (data not shown). In contrast, one-way ANOVA of the proteasome core P20S subunits α + β levels showed significantly decreased levels with aging (F_(3,10)_ = 5.290, *p* = 0.0192). Tukey’s HSD Test for multiple comparisons verified P20S α + β levels significantly reduced when compared group 1 with group 3 (*p* < 0.05) and group 4 (*p* < 0.05) (Figure 6A).

Total GSK3α/β protein levels showed a tendency to decrease with age. A one-way ANOVA revealed significant differences in GSK3α/β protein levels between at least two groups (F_(3,9)_ = 5.406, *p* = 0.0211). Tukey’s post-hoc test validated reduction in GSK3α/β protein levels in the group 4 when compared with group 1 (Figure 6A).

Protein levels of mitochondrial membrane VDAC and ATP synthase, and autophagy (ATG5) were also assessed by western blotting. One-way ANOVA disclosed no significant modifications in VDAC (F_(3,10)_ = 2.054, *p* = 0.1703) and ATP synthase (F_(3,10)_ = 0.7922, *p* = 0.5255). ATG5 protein levels were not modified with age (F_(3,10)_ = 1.739, *p* = 0.2220) (data not shown).

The AT8 antibody did not detect immunoreactivity in any case excepting one rare case in group 1 (data not shown). The rare case was not accompanied by AT8 immunoreactivity in histological sections, and the reason of AT8 positivity remains unknown.

ERMN, VPS26, and P20S α + β protein levels were correlated with age (*p* = 0.0015, *p* = 0.0191 and *p* = 0.0132, respectively) (Figure 6B). The small number of cases per group (even lower than the number used for phosphoproteomics) does not permit robust conclusions. Antibodies directed against deregulated phosphoproteins identified in the present study were not available. Therefore, no attempt was made to validate (phospho)proteomics observations.

## DISCUSSION

The American Academy of Neurology estimates that MCI is present in about 8% of people aged 65 to 69, 15% of 75- to 79-year-olds, 25% of those aged 80 to 84, and about 37% of people older than 85 years of age. About 7.5% will develop dementia in the first year after diagnosis of MCI; about 15% will develop dementia in the second year; one-third will develop dementia due to sAD within five years. Between 25% and 50% of individuals over the age of 85 have dementia, most of them due to AD [48–50]. Most people at the age of 65 have ADNC at early NFT stages (I–III) without cognitive impairment; they are categorized as “normal brain aging” and accepted as “controls” in most clinical and pathological studies. However, only about 1% of individuals at the age 80/85 do not have ADNC [1, 2, 25].

Previous (phospho)proteomics studies in human brain aging have been conducted on human brain samples at different stages of NFT and SP pathology, including cases with first stages of ADNC (named “controls”) and subjects with middle and advanced stages of sAD. The present study used brain samples of individuals without neurological deficits and without NFTs and SPs in any region of the telencephalon (ABC score 0/0/0); moreover, cases did not have any other neuropathological change including TDP-43 proteinopathy (LATE), α-synucleinopathy, other tauopathies such as argyrophilic grain disease, frontotemporal lobar degeneration (FTLD), hippocampal sclerosis, and vascular diseases. We chose the FC because of its role in cognition and emotion and the abundant molecular information that permits comparison with other studies.

The primary specific limitation of the present approach is the small number of cases (*n* = 4 for every group). This is due to the rarity to find late-elderly individuals with no ADNC and without any other neuropathological lesion [51]. Due to their size, molecular properties, and methodological restraints, the limited capacity to detect numbers of proteins is a general curtailment of (phospho)proteomics studies, as well. Yet, the present observations have shown changes in the expression levels of a large number of proteins and the phosphorylation state, either hyper- or hypo-phosphorylation, of numerous proteins throughout brain aging: 308 differential expressed proteins and 306 phosphoproteins were identified when comparing all age groups. Notably, 280 differentially expressed proteins and 278 phosphopeptides were familiar to the four groups. Changes were not related to sex differences.

### Human FC proteome with aging

Previous proteomic studies in human brain aging were focused on the identification protein pathways associated with cognitive decline and dementia [3–18]. Most of them analyzed different brain regions including the hippocampus, temporal cortex, and cerebellum not covered in the present study; and others examined the prefrontal cortex [8, 11, 12, 14]. One interesting study revealed two different pathways involved in Alzheimer’s dementia; one affecting β-amyloid deposition and another affecting resilience without a known pathological footprint [8]. Other studies stressed regional differences in the proteome profiles and linked cognitive impairment with altered expression of several cytoskeletal proteins [11, 14]. The prefrontal cortex was analyzed at early and advanced stages of NFT pathology, showing significant modifications in the level of proteins involved in catabolic processes, mRNA splicing, integrin-mediated signaling pathway, cytoskeleton, and synapse related-proteins at stages I-II [14]. In the same study, alterations in the expression of many other proteins occurred with disease progression [14]. Another study revealed mitochondrial protein alterations already present at early stages of AD that increased at advanced stages of the disease [12].

In the present study, proteins with altered expression levels in the FC during brain aging are components of the cytoskeleton, membranes, synapses, vesicles, myelin, and proteins linked to membrane transport and ion channels. Others connect with DNA and RNA metabolism, ubiquitin-proteasome-system (UPS), kinases, and phosphatases. The remaining abnormally expressed proteins participate in fatty acid metabolism, mitochondria, and GTPase functions. Therefore, our results fill the gap between brain ageing without ADNC, and cases with early and advanced stages of AD pathology. The relevant contribution of the present study deals with the identification of proteome alterations in the aged human frontal cortex in individuals with no neurological deficits, and without ADNC and other age-related neuropathological lesions.

The present study shows relative stable levels of proteins although with individual variations probably linked to the limited number of cases per group in the human FC in the two major clusters (named 1 and 7) until the age of 70. However, protein levels of functional components of cell membranes, vesicles and synapses, RNA modulation, and cellular structures (including tau and tubulin filaments) are markedly altered in the four individuals aged 75 or more. Furthermore, main alterations in the proteome are associated with proteins specific to neuronal populations, rather than those found in other cell types in the brain.

Comparison of these results with other published observations in AD cases identifies particular profiles in cases without ADNC. In AD, clathrin-mediated endocytosis decreases [52] whereas clathrin increases in non-ADNC cases. ERMN is an actin filament binding activity, involved in actin filament organization, mainly localized in oligodendroglia; decreased expression is in line with altered cytoskeleton with aging [53].

Damaged proteins are degraded by the ubiquitin-proteasome system which is key component of the proteostasis network. Proteasomal dysfunction is associated with an increased risk of protein aggregation, chronic inflammation, and the development of age-related diseases [54, 55]. Here, we analysed the protein levels of the 19-subunit regulatory particle, that recognizes substrates via a polyubiquitin tag [56], the immunoproteasome components LMP2 and LMP7 subunits [57], and the P20S α + β proteasome core [58]. The present findings show decreased expression of P20S α + β and preserved expression of P19S and immunoproteasome subunits LMP2 and LMP7 with aging. The relative preservation of the proteasome may act as a resilience mechanism in advanced aging protecting cells from the accumulation of altered proteins [55, 59–62].

Our preliminary observations assessing expression levels of ATG5, a crucial autophagy component, indicate no changes with age which contrast with the expected decline in aging-related neurological disorders [63]. Protein levels of some mitochondrial membranes were also altered at advanced ages in the proteomics study; however, VDAC and ATP synthase levels were preserved in western. It is contrast with mitochondrial dysfunction observed in aging and aging-related diseases [64]. The maintenance of these markers may indicate preservation of these pathways, in contrast to the observations in neurodegenerative disorders [65–72].

Finally, in addition to these results, we observed reduced levels of GSK3α/β. GSK3α/β is considered a key player in the pathophysiology of different age-related brain diseases since dysregulation of this kinase influences protein phosphorylation, neuroinflammation, neurogenesis, and alteration of synaptic function and memory, among others [73]. GSK3β is found to be hyperactive in the brains of AD patients [74], and its expression levels are known to increase with age [75]. Reduced levels of GSK3α/β may be understood as protective [76–80], and are in line with partial preservation of the proteasome, which is able to maintain GSK3β levels reduced [76].

However, the present study was carried out in the frontal cortex; therefore, we cannot rule out the possibility that additional or other changes may occur in different brain regions with aging.

Aged mice do not have ADNC; therefore, quantitative proteomics in the murine brain during aging provides non-biased information. Main alterations in old mice involve synaptic transmission, cytoskeleton, mitochondria and energy metabolism, oxidative stress, ribosome, transcriptional regulation, and GTPase function [81–83]. Therefore, proteome changes identified in the aged human frontal cortex are similar to those reported in the cerebral cortex of the aged murine brain.

### Human FC phosphoproteome with aging

The individual categorization of deregulated phosphoproteins in the present study identifies many structural components of the cytoskeleton, including microfilaments, actin-binding proteins, intermediate filaments of neurons and glial cells, and microtubules; and phosphoproteins of the membranes, synapses, and dense core vesicles. Other deregulated phosphoproteins are kinases and phosphatases, proteins linked to DNA and RNA, and components of the UPS, phosphoproteins involved in GTPase regulation, inflammation, and lipid metabolism. Interestingly, only a few deregulated phosphoproteins also show altered expression levels, thus implying that protein expression levels and phosphorylation are modulated differently during human brain aging.

As indicated before, previous phosphoproteomics studies in human brain aging are representative of the phosphoproteome at different stages of ADNC including sAD [10, 32–37, 39]. Although with variations from one study to another, main deregulated phosphoproteins were associated with integral membrane proteins, glycoproteins, cytoskeletal proteins, synapsis, metabotropic glutamate receptor 5, calcium-signalling pathways, small heat shock proteins (HSP-27 and crystallin-αB), serine/threonine kinases, and mRNA processing and splicing [36, 37, 39].

In a previous study, the total number of identified deregulated phosphoproteins in the human FC in individuals with ADNC was 167, corresponding to 81 at NFT stages I-II, 92 at NFT stages III-IV, and 79 at NFT stages V-VI when compared with control cases without NFT and SP pathology [38]. The main group of deregulated phosphoproteins throughout sAD progression was composed of membrane proteins, proteins of the cytoskeleton, proteins of the synapses and dense core vesicles, and proteins linked to membrane transport and ion channels. Other deregulated phosphoproteins were kinases, proteins connected to DNA or protein deacetylation, proteins related to gene transcription and protein synthesis, heat-shock proteins, members of the UPS, and proteins involved in energy metabolism [38].

The control group (group 1) in the present study on brain aging without ADNC was aged 30–44, whereas the control group in our previous study was aged 33–79 years. Therefore, these control groups cannot be used as shared “controls”. Consequently, comparisons between the present findings and those of previous (phospho)proteomics studies are only approximate. Similarly to the observations in the proteome, changes in the phosphoproteome in the human FC show little variations until the age of 70. However, marked modifications occur in the larger phosphoprotein clusters 4 and 8 involving the cytoskeleton and neuronal structures, membrane stabilization, and kinase regulation in the late-elderly. Consistent with the observations in proteomic data, the analysis of altered phosphoproteins in cell populations revealed that the changes are mainly linked to neurons rather than to other brain cell types.

Considering phosphoproteome modifications in GO terms associated with cell functions and sub-cellular localization, the primary identified affected pathways are similar in brain aging without ADNC and progressive stages of sAD. However, main alterations in sAD and related murine models appear at the first stages of the disease and augment at the middle stages [38, 44]. Moreover, they involve particular systems linked to membrane proteins, membrane signalling, synapses, and cytoskeleton, in conjunction with activation of specific kinases involved in tau phosphorylation [38].

Similar phosphoproteomes are identified in other human and mouse tauopathies, although with disease-specific proteins [40, 41, 47]. A common unifying (phospho)protein in sAD and tauopathies is the early aberrant phosphorylation of tau with variable involvement of phosphorylation sites. The association of tau with the plasma membrane is determined by its phosphorylation pattern, and the phosphorylation state of membrane proteins, proteins linked to membrane signaling, and membrane specializations, together with the lipid composition of membranes, modulate tau phosphorylation [84–90]. Differences between present series without ADNC, and AD and tauopathies appear to be related to key modifications of membrane proteins, membrane signaling, and massive activation of specific tau kinases.

Altered phosphorylation occurs at early stages in other human neurodegenerative diseases and related mouse models [91–93]. Protein phosphorylation deregulation, involving cAMP signaling, dendrite development, and microtubule binding, precedes and extends pathology beyond the mutated polyglutamine tract in the cerebral cortex of Huntington’s disease transgenic mice [93]. Phosphoproteomics has also revealed that aberrant p25/Cdk5 signaling occurs in early-stage Parkinson’s disease in α-synuclein transgenic mice [92], whereas PINK1 regulates a subset of Rab GTPases [94]. The ultimate reason for the selective disease-specific altered protein phosphorylation remains unsolved. However, deregulated kinases play a principal role; the application of computational algorithms on phosphoproteomic data may permit the inference of kinase activity, facilitating the identification of deregulated kinases in various diseases [95].

Therefore, deregulated protein phosphorylation is universal in human brain aging and neurodegenerative diseases, but target proteins and molecular pathways involved are disease-dependent. The present observations identify proteostatic changes, including different changes in the phosphoproteome in the human FC in brain aging in the rare subpopulation of old-aged individuals without neurological deficits, and not having ADNC and other neuropathological change in any region of the telencephalon.

## MATERIALS AND METHODS

### Human tissue samples

Donors included in the present study did not have neurological and mental complains, particularly they did not suffer from cognitive impairment, and they were able to carry out daily activities. The neurological examination at the time of admission in the hospital revealed no alterations. Causes of admission were variable including cardiac and respiratory diseases, infectious diseases, and malignancies. The causes of death were also variable and were attributed to cardiac, respiratory and multi-systemic failure.

The frozen frontal cortex of post-mortem samples was obtained from the Institute of Neuropathology HUB-ICO-IDIBELL Biobank following the guidelines of Spanish legislation on this matter and the approval of the local ethics committee (CEIC) of Bellvitge University Hospital. The post-mortem interval between death and tissue processing was between 2 h 45 min and 21 h. One hemisphere was immediately cut in coronal sections, 1 cm thick, and selected areas of the encephalon were rapidly dissected, frozen on metal plates over dry ice, placed in individual air-tight plastic bags, numbered with water-resistant ink, and stored at −80°C until used for biochemical studies. The other hemisphere was fixed by immersion in 4% buffered formalin for three weeks for morphologic study. The neuropathological study was carried out on paraffin sections of twenty-five selected regions of the cerebrum, cerebellum, brain stem, and spinal cord, which were stained with hematoxylin and eosin, Klüver-Barrera, and periodic acid Schiff, or processed for immunohistochemistry with anti-β-amyloid, phospho-tau (clone AT8), α-synuclein, αB-crystallin, neurofilament, internexin, TDP-43, TDP-43-P, ubiquitin, p62, glial fibrillary acidic protein, CD68, and IBA1 antibodies. Cases were selected from medium-class Caucasian individuals living in the city with no neurological and mental disorders and dying in the hospital due to distinct disorders mainly systemic neoplasia and infectious disease not affecting the nervous system.

The neuropathological examination revealed no AD-neuropathological changes (neurofibrillary tangles and senile plaques) in any region of the telencephalon (ABC score 0/0/0). Selected cases did not suffer from tauopathy, α-synucleinopathy, TDP-43 proteinopathy, frontotemporal lobar degeneration (FTLD), other neurodegenerative diseases, and vascular diseases affecting the nervous system excepting mild small blood vessel disease. In short, all cases were free from neurological and neuropathological disease. The cases were classified by age (years) in four groups (*n* = 4 cases/group): group 1 (young): 30–44; group 2 (middle-aged: MA): 45–52; group 3 (early-elderly): 64–70; and group 4 (late-elderly): 75–85. A summary of cases is shown in Table 1. The frontal cortex area 8 (and not the hippocampus) was chosen because of its role in cognition and emotion and the abundant molecular information that permits comparison with other studies.

**Table 1:** List of cases used in the analysis of the frontal cortex (phospho)proteome and Western blotting (WB) validation.

**Case**	**Sex**	**Age**	**Group**	**PMD**
1	M	30	Group 1: young	4 h 10 min
2	M	36		2 h 45 min
3	M	39		3 h 30 min
4	M	44		6 h 40 min
5	M	45	Group 2: middle-aged	4 h 05 min
6	F	46		7 h 15 min
7	M	47		4 h 55 min
8	M	52		4 h 40 min
9	M	64	Group 3: early-elderly	3 h 30 min
10	F	66		8 h 00 min
11	M	67		5 h 00 min
12	M	70		13 h 00 min
13	F	75	Group 4: late-elderly	3 h 00 min
14	M	79		7 h 00 min
15	F	80		21 h 00 min
16	M	85		5 h 45 min

Abbreviation: PMD: post-mortem delay. Sample 1 was not available for WB.

### Phosphoproteomic analysis

FC samples were homogenized in a lysis buffer containing 7 M urea, 2 M thiourea, and 50 mM DTT supplemented with protease and phosphatase inhibitors. The High-Select^™^ TiO_2_ Phosphopeptide Enrichment Kit (Thermo Scientific, Barcelona, Spain) was used to obtain the phosphorylated peptide fractions, according to the manufacturer’s instructions. Protein extraction, in-solution digestion (600 μg), peptide purification, and reconstitution before mass spectrometric analysis were performed as previously described [38]. Prior to phosphopeptide enrichment, 10 μg of protein digest were separated for complete proteome analysis.

Phospho-peptide enriched and non-enriched (full proteome analysis) samples were analyzed by an LC-MS/MS Ultimate 3000 UHLPC System coupled to an Exploris480 mass spectrometer (Thermo Scientific, San Jose, CA, USA), by data-dependent analysis (DDA). Peptides were resolved using an C18 Acclaim PepMap 100 trap-column (100 μm × 2 cm, 5 μm, 100Å) and C18 Acclaim PepMap 100 column (75 μm × 500 mm, 2 μm, 100 Å; Thermo Scientific), using a 120-min gradient, at flow rate of 300 μl/min (40^o^C), consisting in: 5% to 20% B in 100 min, 20% to 32% B in 20 min and 32% to 90% B in 1 min (A = Formic 0.1%; B = Acetonitrile). DDA was set using many scans per duty cycle mode (*n* = 20). MS1-survey spectra were measured with a resolution of 120,000 (AGC target: 300%; maximum injection time: 60 ms; mass range: from 320 to 1500 m/z). After the survey scan, tandem MS was performed on the most abundant precursors (Isolation window: 1.4 m/z; charge state: + 2–6; collision energy: 30%). The resulting fragments were detected with a resolution of 30000 (First mass: 110 m/z; AGC target for MS/MS: 100%; maximum injection time: 60 ms). Dynamic exclusion was set to 30 sec with a 10 ppm mass tolerance around the precursor and its isotopes.

Raw files were processed with MaxQuant v 2.0.1 using the integrated Andromeda Search engine [96]. All data were searched against a target/decoy version of the Human Uniprot Reference Proteome with isoforms (Proteome ID: UP000005640_9606; March 2021). The first search peptide tolerance was set to 20 ppm, and the primary search peptide tolerance was set to 4.5 ppm. Fragment mass tolerance was set to 20 ppm. Trypsin was specified as the enzyme cleaving after all lysine and arginine residues allowing up to two missed cleavages. Carbamidomethylation of cysteine was defined as fixed modification, and peptide N-terminal acetylation, oxidation of methionine, deamidation of asparagine, glutamine and pyro-glutamate formation from glutamine and glutamate, and phosphorylation of serine, threonine, and tyrosine were considered variable modifications, with a total of 2 variable modifications per peptide. “Maximum peptide mass” was set to 7,500 Da, the “modified peptide minimum score” and “unmodified peptide minimum score” were set to 25, and everything else was put to the default values, including the false discovery rate limit of 1% on both the peptide and protein levels.

The Perseus software (version 1.6.14.0) was used for statistical analysis and data visualization [97]. One-way ANOVA test was applied to compare data between groups. Only (phospho)peptides with a *p*-value < 0.05 were considered differentially expressed. Proteomic experiments generate a large number of peptide or proteins that need to be evaluated independently using statistical tests maybe yielding type I errors [98]. However, it is important to note that due to the often-low power of proteomic experiments, the use of these corrections may fail to detect even true positives [99]. In this case, the use of only four samples per group, together with the low fold changes quantified in our data, were determinant for the statistical analysis; in consequence, the use of False Discovery Rate (FDR) corrections was not helpful in detecting significant phosphopeptides. MS data and search results files were deposited in the Proteome Xchange Consortium via the JPOST partner repository (https://repository.jpostdb.org) [100] with the identifier PXD035997 for ProteomeXchange and JPST001814 for jPOST (for reviewers: https://repository.jpostdb.org/preview/78674985762f6283a7330b; Access key: 8154).

### Bioinformatics

The identification of significantly dysregulated regulatory/metabolic pathways in FC proteomic datasets was made using Metascape [101]. A cell-type enrichment analysis across frontal cortex differential datasets was performed using cell-type protein marker lists derived from four purified brain cell types: neuron, astrocyte, microglia, oligodendrocyte and endothelial cells [102, 103]. Functional analysis of altered clusters of proteins was performed using ShinyGO (v0.77) software [104].

### Gel electrophoresis and immunoblotting

Frozen samples of frontal cortex area 8 at different patient ages (*n* = 14) were homogenized in RIPA lysis buffer composed of 50 mM Tris/HCl buffer, pH 7.4 containing 2 mM EDTA, 0.2% Nonidet P-40, 1 mM PMSF, protease and phosphatase inhibitor cocktail (Roche Molecular Systems, USA). The homogenates were centrifuged for 20 min at 20,000 × g. Protein concentration was determined with the BCA method (Thermo Scientific). Equal amounts of protein (12 μg) for each sample were loaded and separated by electrophoresis on 10% sodium dodecyl sulfate-polyacrylamide gel electrophoresis (SDS-PAGE) gels and then transferred onto nitrocellulose membranes (Amersham, Freiburg, GE). Non-specific bindings were blocked by incubating 3% albumin in PBS containing 0.2% Tween for one h at room temperature. After washing, the membranes were incubated overnight at 4°C with antibodies against primary antibodies summarized in Table 2. Protein loading was monitored using an antibody against β-actin. Membranes were incubated for one h with appropriate HRP-conjugated secondary antibodies (1:2,000, Dako, Barcelona, Spain); the immunoreaction was visualized with a chemiluminescence reagent (ECL, Amersham). Densitometric quantification was performed using the ImageLab v4.5.2 software (BioRad), using β-actin for normalization.

**Table 2:** List of antibodies used for Western blotting and immunohistochemistry.

**Antibody**	**Supplier**	**References**	**Species**	**Dil. WB**
Clathrin (heavy chain)	BD Transduction	610499	Ms	1/1,000
Ermin (ERMN)	Abcam	ab243730	Rb	1/1,000
SNAP25	Chemicon	MAB331	Ms	½,000
β-tubulin III	Signalway Antibody	21617	Rb	1/1,000
Vimentin	Abcam	ab137321	Rb	1/3,000
VPS26	Genetex	GTX106297	Rb	½,000
β-actin	Sigma	A5316	Ms	1/30,000
AT8	Innogenetics	90206	Ms	1/1,000
P20S α+β	Abcam	ab22673	Rb	1/1,1000
P19S	Abcam	ab3317	Rb	1/1,000
P20S LMP2	Abcam	ab3328	Rb	1/500
P20S LMP7	Abcam	ab3329	Rb	1/500
ATG5	Cell Signaling	#12994	Rb	1/1,000
GSK3α/β	Santa Cruz Biotech.	SC7291	Ms	1/1,000
VDAC	Abcam	ab15895	Rb	1/500
ATP synthase	Biosciences	612516	Ms	1/10,000

Abbreviations: Ms: mouse monoclonal; Rb: rabbit polyclonal; Dil. WB: dilution Western blotting. BD Transduction: http://www.bdbiosciences.com; Abcam: https://www.abcam.com; Chemicon: https://chemicongroup.com; Signalway: https://www.sabbiotech.com; Genetex: https://www.genetex.com; Sigma: https://www.sigmaaldrich.com.

### Statistical analysis

The normality of distribution was analyzed with the Kolmogorov-Smirnov test. The statistical analysis of protein levels between groups was carried out using a one-way analysis of variance (ANOVA) followed by a Tukey post-test using the GraphPad Prism software version 9.5.0 (La Jolla, CA, USA). Graphic design was performed with GraphPad Prism version 9.5.0 (La Jolla, CA, USA). Outliers were detected using the GraphPad software QuickCalcs (*p* < 0.05). Data were expressed as mean ± SEM. When comparing between age groups, differences were considered statistically significant when compared one age group (for example late-elderly (group 4) with young (group 1) at ^*^p < 0.05, ^**^p < 0.01, ^***^p < 0.001; with MA (group 2) at ^#^*p* < 0.05, and ^##^*p* < 0.01; and with early-elderly (group 3) at ^$^*p* < 0.05, and ^$$^*p* < 0.01. Pearson’s correlation coefficient was used to assess associations between protein levels and age. Pearson’s correlation significance levels were set at ^*^p < 0.05, ^**^p < 0.01, and ^***^p < 0.001.

### Data availability statement

All data are available in the main manuscript, and supplementary Figures and Tables.

## Supplementary Materials

Supplementary Figure 1

Supplementary Table 1

Supplementary Tables 2-13
