# Supplementary material for: Proteostatic modulation in brain aging without associated Alzheimer’s disease-and age-related neuropathological changes
Source: Aging (Albany NY). 2023 May 13;15(9):3295–330. doi: 10.18632/aging.204698 (PMC10449282; doi:10.18632/aging.204698)
Supplement: Supplementary Figure 1 [file aging-15-204698-s001.pdf]

SUPPLEMENTARY FIGURE

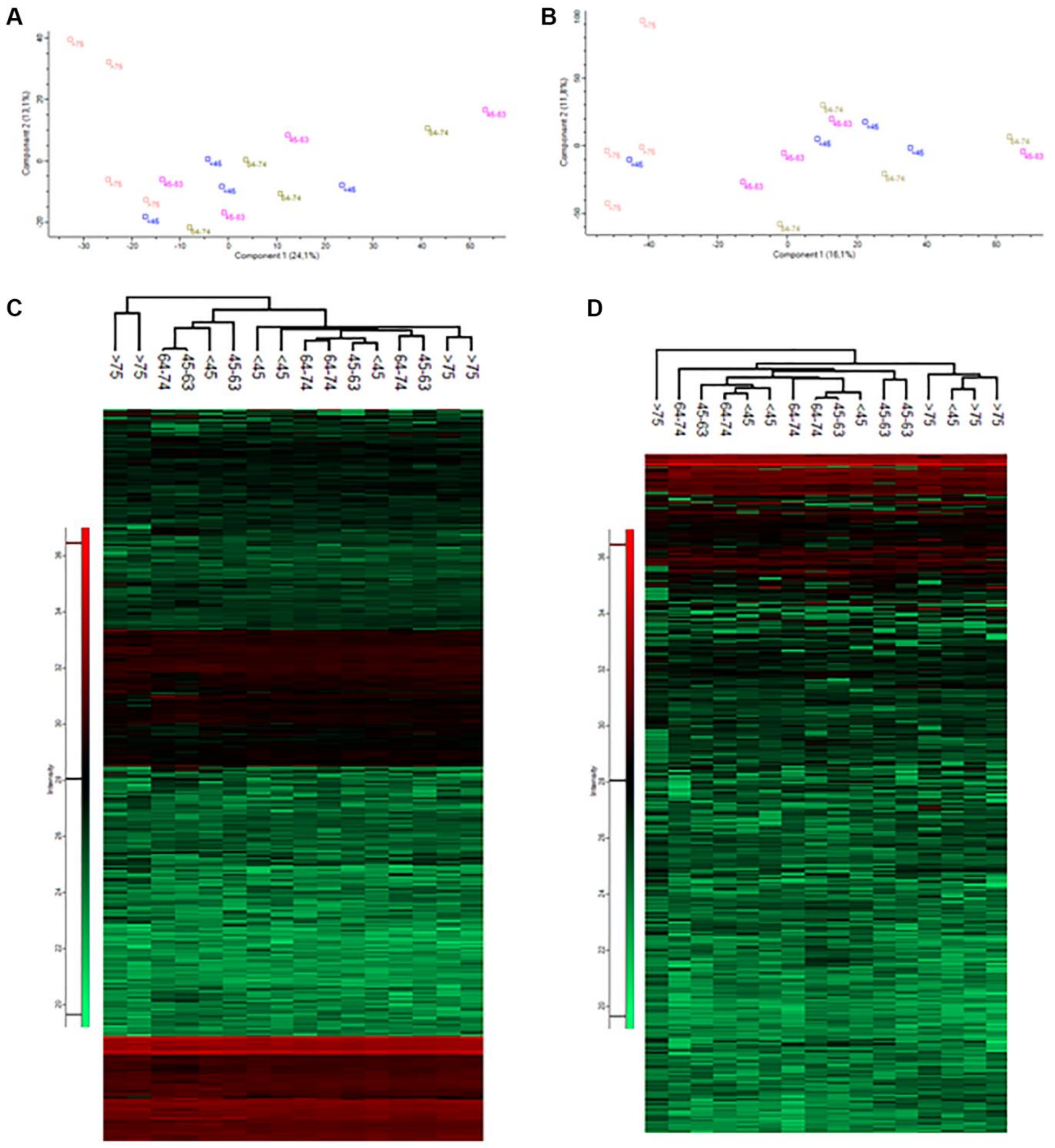

Supplementary Figure 1. Proteomic and phosphoproteomic global analysis. (**A**, **B**) Principal component (PCA) analysis representing all 16 samples at the proteome (**A**) and phosphoproteome (**B**) levels. (**C**, **D**) Heatmaps representing the total number of quantified proteins at the proteome (**C**) and phosphoproteome (**D**) across all age groups.
