# Supplementary material for: Proteostatic modulation in brain aging without associated Alzheimer’s disease-and age-related neuropathological changes
Source: Aging (Albany NY). 2023 May 13;15(9):3295–330. doi: 10.18632/aging.204698 (PMC10449282; doi:10.18632/aging.204698)
Supplement: Supplementary Tables 2-13 [file aging-15-204698-s003.pdf]

SUPPLEMENTARY TABLES

Supplementary Table 2. Abnormally-regulated kinases and phosphatases in proteome across age groups.

Protein ID	Protein name	Function
AGK	Acylglycerol Kinase	Kinase - lipid metabolism
AK3	Adenylate Kinase 3	Mitochondrial kinase
CAMK1	Calcium/Calmodulin Dependent Protein Kinase I	Calcium dependent kinase
CMPK1	Cytidine/Uridine Monophosphate Kinase 1	Kinase activity
CRKL	CRK Like Proto-Oncogene, Adaptor Protein	Kinase activity
DCLK1	Doublecortin Like Kinase 1	Cytoskeleton regulator
FCSK	Fucose Kinase	Cell-cell signaling
FN3KRP	Fructosamine 3 Kinase Related Protein	Kinase with multiple targets
GSK3B	Glycogen Synthase Kinase 3 Beta	Kinase with multiple targets
KALRN	Kalirin RhoGEF Kinase	Cytoskeletal regulator
MAP4K2	Mitogen-Activated Protein Kinase Kinase Kinase Kinase 2	Kinase with multiple targets
NME1	NME/NM23 Nucleoside Diphosphate Kinase 1	Purine metabolism
PAK6	P21 (RAC1) Activated Kinase 6	Cytoskeleton regulator
PFKM	Phosphofructokinase, Muscle	Glycolysis kinase
PFKP	Phosphofructokinase, Platelet	Glycolysis kinase
PRKCA	Protein Kinase C Alpha	Kinase with multiple targets
PRKRA1B	Protein Kinase CAMP-Dependent Type I Regulatory Beta	Kinase with multiple targets
SKP1	S-Phase Kinase Associated Protein 1	UPS kinase
STK24	Serine/Threonine Kinase 24	Cytoskeleton regulator
STRAP	Serine/Threonine Kinase Receptor Associated Protein	RNA metabolism
TAOK1	TAO Kinase 1	Cytoskeleton regulator
TKFC	Triokinase And FMN Cyclase	Kinase with multiple targets
ACP1	Acid Phosphatase 1	Phosphatase
CPPED1	Calcineurin Like Phosphoesterase Domain Containing 1	Phosphatase
ENOPH1	Enolase-Phosphatase 1	Phosphatase/Enolase
PGP	Phosphoglycolate Phosphatase	Phosphatase
PPME1	Protein phosphatase methylesterase 1 (PME-1)	Phosphatase
PPP1CA	Protein Phosphatase 1 Catalytic Subunit Alpha	Phosphatase
PPP1R14A	Protein Phosphatase 1 Regulatory Inhibitor Subunit 14A	Phosphatase
PPP2R2D	Protein Phosphatase 2 Regulatory Subunit Bdelta	Phosphatase
PPP6C	Protein Phosphatase 6 Catalytic Subunit	Phosphatase

Supplementary Table 3. Abnormally-regulated cytoskeleton-related proteins in proteome across age groups.

Protein ID	Protein name	Function
ABL2	ABL Proto-Oncogene 2, Non-Receptor Tyrosine Kinase	Cytoskeleton regulator
ACTG1	Actin Gamma 1	Cytoskeletal component
AGAP1	ArfGAP With GTPase Domain, Ankyrin Repeat and PH 1	Cytoskeletal component
AGAP2	ArfGAP With GTPase Domain, Ankyrin Repeat and PH 2	Cytoskeletal component
ARR3	Arrestin 3	Cytoskeletal component
BGN	Biglycan	ECM component
CAP2	Cyclase Associated Actin Cytoskeleton Regulatory Protein 2	Cytoskeleton regulator
CKAP5	Cytoskeleton Associated Protein 5	Cytoskeletal component
CLASP2	Cytoplasmic Linker Associated Protein 2	Cytoskeletal component
CORO1C	Coronin 1C	Cytoskeletal component
CTTN	Cortactin	Cytoskeletal regulator
DNM3	Dynamin 3	Cytoskeletal component
DYNLL2	Dynein Light Chain LC8-Type 2	Cytoskeletal component
ERLIN2	ER Lipid Raft Associated 2	Cytoskeletal regulator
ERMN	Ermin	Cytoskeletal component
FMNL2	Formin Like 2	Cytoskeletal regulator

FMOD	Fibromodulin	ECM component
GAP43	Growth Associated Protein 43	Cytoskeletal component
GMFB	Glia Maturation Factor Beta	Cytoskeletal component
HIP1R	Huntingtin Interacting Protein 1 Related	Cytoskeletal component
KIF2A	Kinesin Family Member 2A	Cytoskeletal component
KIF3B	Kinesin Family Member 3B	Cytoskeletal component
KLC1	Kinesin Light Chain 1	Cytoskeletal component
KLK6	Kallikrein Related Peptidase 6	ECM regulator
LMNB2	Lamin B2	Cytoskeletal component
LUM	Lumican	ECM component
MAP1LC3A	Microtubule Associated Protein 1 Light Chain 3 Alpha	Cytoskeletal component
MAP7D1	MAP7 Domain Containing 1	Cytoskeletal component
MTPN	Myotrophin	Cytoskeletal component
MYL1	Myosin Light Chain 1	Cytoskeletal component
NCK2	NCK Adaptor Protein 2	Cytoskeletal regulator
NEBL	Nebulette	Cytoskeletal component
NUDC	Nuclear Distribution C, Dynein Complex Regulator	Cytoskeletal regulator
NUDCD2	NudC Domain Containing 2	Cytoskeletal regulator
PADI2	Peptidyl Arginine Deiminase 2	Cytoskeletal regulator
PKP2	Plakophilin 2	Cytoskeleton component
PRELP	Proline And Arginine Rich End Leucine Rich Repeat Protein	ECM component
RIC8A	RIC8 Guanine Nucleotide Exchange Factor A	Cytoskeletal regulator
ROCK2	Rho Associated Coiled-Coil Containing Protein Kinase 2	Cytoskeletal regulator
SHTN1	Shootin 1	Cytoskeletal regulator
STMN1	Stathmin 1	Cytoskeletal regulator
TNS3	Tensin 3	Cytoskeletal component
TPPP	Tubulin Polymerization Promoting Protein	Cytoskeletal component
TRIM3	Tripartite Motif Containing 3	Cytoskeletal component
TUBB2A	Tubulin Beta 2A Class Iia	Cytoskeletal component
TUBB3	Tubulin Beta 3 Class III	Cytoskeletal component
VIM	Vimentin	Cytoskeletal component

Supplementary Table 4. Abnormally-regulated membrane-related proteins in proteome across age groups.

Protein ID	Protein name	Function
ABCA2	ATP Binding Cassette Subfamily A Member 2	Membrane transporter protein
ABCC4	ATP Binding Cassette Subfamily C Member 4	Membrane transporter protein
ADAM11	ADAM Metallopeptidase Domain 11	Membrane protein/Cell adhesion
AGFG1	ArfGAP With FG Repeats 1	Vesicles membrane
ANKHD1	Ankyrin Repeat and KH Domain Containing 1	Integral membrane protein
ANXA2	Annexin A2	Membrane component
ANXA4	Annexin A4	Membrane component
APBA2	Amyloid Beta Precursor Protein Binding Family A 2	Vesicular trafficking protein
ATP1A3	ATPase Na+/K+ Transporting Subunit Alpha 3	Membrane transporter
ATP5F1E	ATP Synthase F1 Subunit Epsilon	Membrane transporter
ATP6V1A	ATPase H+ Transporting V1 Subunit A	Membrane transporter
ATP6V1B2	ATPase H+ Transporting V1 Subunit B2	Membrane transporter
BASP1	Brain acid soluble protein 1	Membrane component
BCAS1	Brain Enriched Myelin Associated Protein 1	Membrane protein
BSG	Basigin	Membrane protein
CACNB4	Calcium Voltage-Gated Channel Auxiliary Subunit Beta 4	Membrane ion channel
CD9	CD9 Molecule	Membrane component/cell junction
CHCHD3	Coiled-Coil-Helix-Coiled-Coil-Helix Domain Containing 3	Integral membrane protein
CHCHD6	Coiled-Coil-Helix-Coiled-Coil-Helix Domain Containing 6	Integral membrane protein
CLDN11	Claudin 11	Integral membrane protein

CLTB	Clathrin Light Chain B	Vesicle membrane protein
CLTC	Clathrin Heavy Chain	Vesicle membrane protein
CNTNAP2	Contactin Associated Protein 2	Membrane component/cell junction
COPS3	COP9 Signalosome Subunit 3	Vesicle membrane protein
COPS4	COP9 Signalosome Subunit 4	Vesicle membrane protein
COPS8	COP9 Signalosome Subunit 8	Vesicle membrane protein
COPZ1	COPI Coat Complex Subunit Zeta 1	Vesicle membrane protein
CTNND1	Catenin Delta 1	Membrane component/cell junction
DNM1L	Dynamin 1 Like	Organelle membrane
EPB41L1	Erythrocyte Membrane Protein Band 4.1 Like 1	Synapse membrane component
EPDR1	Ependymin Related 1	Membrane protein/Cell adhesion
EXOC3	Exocyst Complex Component 3	Vesicles membrane
EXOC7	Exocyst Complex Component 7	Vesicles membrane
EXOC8	Exocyst Complex Component 8	Vesicles membrane
GATD1	Glutamine Amidotransferase Class 1 Domain 1	Vesicles membrane
GBR2	Growth Factor Receptor Bound Protein 2	Membrane component
GLIPR2	GLI pathogenesis-related 2	Organelle membrane
GOPC	Golgi Associated PDZ And Coiled-Coil Motif Containing	Organelle membrane
GOT2	Glutamic-Oxaloacetic Transaminase 2	Mitochondrial membrane protein
GPD1	Glycerol-3-Phosphate Dehydrogenase 1	Mitochondrial membrane protein
GPD2	Glycerol-3-Phosphate Dehydrogenase 2	Mitochondrial membrane protein
IMMT	Inner Membrane Mitochondrial Protein	Mitochondrial membrane protein
JAM2	Junctional Adhesion Molecule 2	Membrane and cell adhesion
LMAN2	Lectin, Mannose Binding 2	Membrane/vesicle protein
MEAK7	MTOR Associated Protein, Eak-7 Homolog	Vesicles membrane
MOG	Myelin Oligodendrocyte Glycoprotein	Membrane protein
NECAP1	NECAP Endocytosis Associated 1	Vesicles membrane
NIPSNAP1	Nipsnap Homolog 1	Vesicles membrane transport
NIPSNAP2	Nipsnap Homolog 2	Vesicles membrane transport
NIPSNAP3B	Nipsnap Homolog 3B	Vesicles membrane transport
OMG	Oligodendrocyte Myelin Glycoprotein	Membrane protein
PGRMC2	Progesterone Receptor Membrane Component 2	Membrane protein
PHB2	Prohibitin 2	Membrane protein
PICALM	Phosphatidylinositol Binding Clathrin Assembly Protein	Vesicles membrane
PMP2	Peripheral Myelin Protein 2	Membrane protein
PPIF	Peptidylprolyl Isomerase F	Mitochondrial membrane protein
RAB12	RAB12 Member RAS Oncogene Family	Vesicles membrane
RAB1B	RAB1B, Member RAS Oncogene Family	Vesicles membrane
RAB21	RAB21, Member RAS Oncogene Family	Vesicles membrane
RABGEF1	RAB Guanine Nucleotide Exchange Factor 1	Vesicles membrane
SLC14A1	Solute Carrier Family 14 Member 1	Membrane transporter
SLC16A7	Solute Carrier Family 16 Member 7	Membrane transporter
SLC24A2	Solute Carrier Family 24 Member 2	Membrane transporter
SLC25A1	Solute Carrier Family 25 Member 1	Membrane transporter
SLC32A1	Solute Carrier Family 32 Member 1	Membrane transporter
SNAP25	Synaptosome Associated Protein 25	Vesicles membrane
SPTAN1	Spectrin Alpha, Non-Erythrocytic 1	Membrane protein/Cytoskeleton
SPTBN1	Spectrin Beta, Non-Erythrocytic 1	Membrane protein/Cytoskeleton
STXBP1	Syntaxin Binding Protein 1	Vesicles membranes
STXBP5L	Syntaxin Binding Protein 5L	Vesicles membranes
THY1	Thy-1 Cell Surface Antigen	Membrane component/Cell junction
TOMM5	Translocase Of Outer Mitochondrial Membrane 5	Membrane protein
UNC13A	Unc-13 Homolog A	Vesicles membrane
VPS13C	Vacuolar Protein Sorting 13 Homolog C	Vesicles membrane

VPS16	VPS16 Core Subunit of CORVET And HOPS Complexes	Vesicles membrane
VPS26A	VPS26, Retromer Complex Component A	Vesicles membrane
YTK6	YTK6 V-SNARE Homolog	Vesicles membrane

Supplementary Table 5. Abnormally-regulated DNA/RNA-related proteins in proteome across age groups.

Protein ID	Protein name	Function
ACIN1	Apoptotic Chromatin Condensation Inducer 1	RNA metabolism
ACO2	Aconitase 2	Glucose metabolism
C11orf68	chromosome 11 open reading frame 68	RNA metabolism
CARHSP1	Calcium Regulated Heat Stable Protein 1	RNA metabolism
DAZAP1	DAZ Associated Protein 1	RNA metabolism
DHX9	DExH-Box Helicase 9	DNA/RNA metabolism
DPY30	Dpy-30 Histone Methyltransferase Complex Regulatory Subunit	DNA regulation
DUT	Deoxyuridine Triphosphatase	DNA metabolism
EDF1	Endothelial Differentiation Related Factor 1	DNA binding
EEF1A2	Eukaryotic Translation Elongation Factor 1 Alpha 2	GTPase/RNA metabolism
EIF2B3	Eukaryotic Translation Initiation Factor 2B Subunit Gamma	GTPase/RNA metabolism
EIF2S3	Eukaryotic Translation Initiation Factor 2 Subunit Gamma	GTPase/RNA metabolism
EIF4A2	Eukaryotic Translation Initiation Factor 4A2	RNA metabolism
EIF4G1	Eukaryotic Translation Initiation Factor 4 Gamma 1	RNA metabolism
ELAVL2	ELAV Like RNA Binding Protein 2	RNA metabolism
EXOG	Exo/Endonuclease G	DNA metabolism
HARS1	Histidyl-TRNA Synthetase 1	RNA metabolism
HNRNPA3	Heterogeneous Nuclear Ribonucleoprotein A3	RNA metabolism
HNRNPH2	Heterogeneous Nuclear Ribonucleoprotein H2	RNA metabolism
HNRNPH3	Heterogeneous Nuclear Ribonucleoprotein H3	RNA metabolism
HNRNPUL2	Heterogeneous Nuclear Ribonucleoprotein U Like 2	RNA metabolism
IFIT1	Interferon Induced Protein with Tetratricopeptide Repeats 1	RNA metabolism
KDM5B	Lysine Demethylase 5B	DNA/Histone
KPNA3	Karyopherin Subunit Alpha 3	DNA/RNA metabolism
KPNA6	Karyopherin Subunit Alpha 6	DNA/RNA metabolism
LSM3	LSM3 Homolog, U6 Small Nuclear RNA Associated	RNA metabolism
LSM8	LSM8 Homolog, U6 Small Nuclear RNA Associated	RNA metabolism
MCTS1	MCTS1 Re-Initiation and Release Factor	RNA metabolism
NONO	Non-POU Domain Containing Octamer Binding	RNA metabolism
PAIP1	Poly(A) Binding Protein Interacting Protein 1	RNA metabolism
PBXIP1	PBX Homeobox Interacting Protein 1	Repressor factor
PRMT1	Protein Arginine Methyltransferase 1	RNA metabolism
PRMT5	Protein Arginine Methyltransferase 5	RNA/DNA metabolism
PROX1	Prospero Homeobox 1	RNA/DNA metabolism
PSPC1	Paraspeckle Component 1	RNA metabolism
PURA	Purine Rich Element Binding Protein A	DNA metabolism
RAD23B	RAD23 Homolog B, Nucleotide Excision Repair Protein	DNA metabolism
RBM14	RNA Binding Motif Protein 14	RNA metabolism
RPL30	Ribosomal Protein L30	RNA metabolism
RPS13	Ribosomal Protein S13	RNA metabolism
RPS16	Ribosomal Protein S16	RNA metabolism
RPS19	Ribosomal Protein S19	RNA metabolism

RTRAF	RNA Transcription, Translation and Transport Factor	RNA metabolism
SSBP1	Single Stranded DNA Binding Protein 1	DNA metabolism
SUGT1	SGT1 Homolog, MIS12 Kinetochore Complex Cochaperone	DNA/RNA metabolism
THAP4	THAP Domain Containing 4	RNA/DNA metabolism
U2AF2	U2 Small Nuclear RNA Auxiliary Factor 2	RNA metabolism
WDR82	WD Repeat Domain 82	DNA metabolism

Supplementary Table 6. Abnormally-regulated UPS proteins in proteome across age groups.

Protein ID	Protein name	Function
CAND1	Cullin-associated NEDD8-dissociated protein 1	UPS
CACYBP	Calcyclin Binding Protein	UPS
DCUN1D1	Defective In Cullin Neddylation 1 Domain Containing 1	UPS
PSMA4	Proteasome 20S Subunit Alpha 4	UPS
PSMC5	Proteasome 26S Subunit, ATPase 5	UPS
PSMD1	Proteasome 26S Subunit, Non-ATPase 1	UPS
PSME3	Proteasome Activator Subunit 3	UPS
TRIM36	Tripartite Motif Containing 36	UPS
UBA52	Ubiquitin A-52 Residue Ribosomal Protein Fusion Product 1	UPS
UBA6	Ubiquitin Like Modifier Activating Enzyme 6	UPS
UBE2I	Ubiquitin Conjugating Enzyme E2 I	UPS
UBE2K	Ubiquitin Conjugating Enzyme E2 K	UPS
UBQLN1	Ubiquilin 1	UPS
UBXN1	UBX Domain Protein 1	UPS
UCHL3	Ubiquitin C-Terminal Hydrolase L3	UPS
UFM1	Ubiquitin Fold Modifier 1	UPS
UFL1	UFM1 Specific Ligase 1	UPS

Supplementary Table 7. Miscellaneous abnormally-regulated proteins in proteome across age groups.

Protein ID	Protein name	Function
ABHD16A	Abhydrolase Domain Containing 16A, Phospholipase	Hydrolase
ACAA1	Acetyl-CoA Acyltransferase 1	Fatty acid metabolism
ACADSB	Acyl-CoA Dehydrogenase Short/Branched Chain	Fatty acid metabolism
ACSF3	Acyl-CoA Synthetase Family Member 3	Fatty acid metabolism
ADO	2-Aminoethanethiol Dioxygenase	Dioxygenase
ADSS2	Adenylosuccinate Synthase 2	Adenylsynthase
AHSA1	Activator Of HSP90 ATPase Activity 1	Co-chaperone
ALDH5A1	Aldehyde Dehydrogenase 5 Family Member A1	GABA metabolism
APOD	Apolipoprotein D	Lipoprotein
APOL2	Apolipoprotein L2	Lipoprotein
APOOL	Apolipoprotein O Like	Lipoprotein
APRT	Adenine Phosphoribosyltransferase	Phophotransferase
ARFIP1	ADP Ribosylation Factor Interacting Protein 1	GTPase regulator
ARL15	ADP Ribosylation Factor Like GTPase 15	GTPase regulator
ARL8A	ADP Ribosylation Factor Like GTPase 8A	GTPase regulator
ASS1	Argininosuccinate Synthase 1	Arginine metabolism
ATG5	Autophagy Related 5	Autophagy
ATL1	Atlastin GTPase 1	GTPase
B4GAT1	Beta-1,4-Glucuronyltransferase 1	Transferase activity
CAB39	Calcium-binding protein 39	Kinase activator

CALM3	Calmodulin 3	Ezymatic cofactor
CARNS1	Carnosine Synthase 1	Synthase
CHDH	Choline Dehydrogenase	Dehydrogenase
COA3	Cytochrome C Oxidase Assembly Factor 3	Mitochondria resp. chain
CRABP1	Cellular Retinoic Acid Binding Protein 1	Carotenoid metabolism
CSE1L	Chromosome Segregation 1 Like	Nuclear transport
CYB5R1	Cytochrome B5 Reductase 1	Fatty acid metabolism
CYCS	Cytochrome C, Somatic	Mitochondria resp. chain
DCXR	Dicarbonyl And L-Xylulose Reductase	Oxidoreductase activity
DDT	D-Dopachrome Tautomerase	Decarboxylase activity
DNAJC7	DnaJ Heat Shock Protein Family (Hsp40) Member C7	Chaperone regulation
F13A1	Coagulation Factor XIII A Chain	Coagulation
FABP5	Fatty Acid Binding Protein 5	Fatty acid metabolism
FABP5	Fatty Acid Binding Protein 5	Fatty acid metabolism
FAM117A	Family With Sequence Similarity 117 Member A	Unknown function
FAM81A	Family With Sequence Similarity 81 Member A	Unknown function
GLRX	Glutaredoxin	Redox system
GMDS	GDP-Mannose 4,6-Dehydratase	Metabolic enzyme
GNA13	G Protein Subunit Alpha 13	GTPase modulator
GNAO1	G Protein Subunit Alpha O1	GTPase modulator
GNB1	G Protein Subunit Beta 1	GTPase activity
GNB2	G Protein Subunit Beta 2	GTPase activity
GSTM1	Glutathione S-Transferase Mu 1	Redox system
GSTO1	Glutathione S-Transferase Omega 1	Redox system
HBA1	Hemoglobin Subunit Alpha 1	Oxygen transport
IDH2	Isocitrate Dehydrogenase (NADP(+)) 2	Mitochondrial protein
LIPE	Lipase E, Hormone Sensitive Type	Lipid metabolism
LRP1	LDL Receptor Related Protein 1	Lipid signaling
LRPAP1	LDL Receptor Related Protein Associated Protein 1	Lipid signaling
LRRC59	Leucine Rich Repeat Containing 59	Nuclear transport
MAT2B	Methionine Adenosyltransferase 2B	Adenosyltransferase
MT-CO1	MT-Encoded Cytochrome C Oxidase I	Mitochondrial protein
MT-ND5	MT-Encoded NADH:Ubiquinone Oxidoreductase Core S. 5	Mitochondrial protein
MTURN	Maturin	Intracellular signaling
MTX2	Metaxin 2	Mitochondrial protein
NDUFAF3	NADH:Ubiq. Oxidoreductase Complex Assembly Factor 3	Mitochondrial protein
NHLRC2	NHL Repeat Containing 2	Unknown function
NIT2	Nitrilase Family Member 2	Histidine metabolism
NLN	Neurolysin	Peptidase
NMT1	N-Myristoyltransferase 1	Fatty acid metabolism
OGN	Osteoglycin	Growth factor
OLA1	Obg like ATPase 1	GTPase
PAFAH1B2	Platelet Activating Factor Acetylhydrolase 1b Catalytic 2	Hydrolase activity
PDCD5	Programmed Cell Death 5	Apoptosis
PEF1	Penta-EF-Hand Domain Containing 1	Complex scaffold protein
PFDN5	Prefoldin Subunit 5	Chaperone
PPT1	Palmitoyl-Protein Thioesterase 1	Fatty acid metabolism
PRXL2B	Peroxiredoxin Like 2B	Reductase
PUDP	Pseudouridine 5'-Phosphatase	Unknown function
RAP1GDS1	Rap1 GTPase-GDP Dissociation Stimulator 1	GTPases
RRAS	RAS related	GTPases
S100B	S100 Calcium Binding Protein B	Cellular signaling
SAR1A	Secretion Associated Ras Related GTPase 1A	GTPase
SDHA	Succinate Dehydrogenase Complex Flavoprotein Subunit A	Mitochondria resp. chain
SDHD	Succinate Dehydrogenase Complex Subunit D	Mitochondria resp. chain
SH3BGRL3	SH3 Domain Binding Glutamate Rich Protein Like 3	GTPase activator

SPR	Sepiapterin Reductase	Reductase
SUB1	SUB1 Regulator of Transcription	Transcription factor
SUCLG2	Succinate-CoA Ligase GDP-Forming Subunit Beta	Glucose metabolism
TXNDC17	Thioredoxin Domain Containing 17	Redox system
YWHAB	Tryptophan 5-Monooxygenase Activation Protein Beta	Monooxygenase activity
YWHAG	Tryptophan 5-Monooxygenase Activation Protein Gamma	Monooxygenase activity
YWHAZ	Tryptophan 5-Monooxygenase Activation Protein Zeta	Monooxygenase activity

Supplementary Table 8. Abnormally-regulated phosphorylated kinases in phosphoproteome across age groups.

Protein ID	Protein name	Function
AATK	Apoptosis Associated Tyrosine Kinase	Kinase
AHNAK	AHNAK Nucleoprotein	Kinase component
Akap12	A-kinase anchor protein 12 (AKAP-12)	Kinase component
BAZ1B	Bromodomain Adjacent to Zinc Finger Domain 1B	Kinase TF
BRSK2	Serine/threonine-protein kinase BRSK2	Cytoskeleton regulator
CAMK1D	Calcium/Calmodulin Dependent Protein Kinase 1D	Calcium dependent kinase
CAMK2A	Calcium/calmodulin-dependent protein kinase type II subunit α	Calcium dependent kinase
CAMK2B	Calcium/calmodulin-dependent protein kinase	Calcium dependent kinase
CAMK2G	Calcium/calmodulin-dependent protein kinase type II subunit γ	Calcium dependent kinase
CAMKV	CaM Kinase Like Vesicle Associated	Vesicle associated kinase
CKB	Creatine Kinase B	Kinase activity
DCLK1	Doublecortin Like Kinase 1	Cytoskeleton regulator
DYRK1A	Dual Specificity Tyrosine Phosphorylation Regulated Kinase 1A	Kinase with multiple targets
KNDC1	Kinase Non-Catalytic C-Lobe Domain Containing 1	Regulation dendrite growth
MINK1	Misshapen Like Kinase 1	Synapse regulation
NUAK1	NUAK Family Kinase 1	Cell adhesion control
PACSIN1	Protein Kinase C And Casein Kinase Substrate in Neurons 1	Cytoskeleton regulator
PAK1	P21 (RAC1) Activated Kinase 1	Cytoskeleton regulator
PI4KA	Phosphatidylinositol 4-Kinase α	Kinase with multiple targets
PIK3C2A	Phosphatidylinositol-4-P-3-Kinase Catalytic Subunit Type 2 α	Kinase with multiple targets
PIP5K1C	Phosphatidylinositol-4-P-5-Kinase Type 1 Gamma	Kinase with multiple targets
PRKCA	Protein Kinase C Alpha	Kinase with multiple targets
PRKCD	Protein Kinase C Delta	Kinase with multiple targets
PRKCG	Protein Kinase C Gamma	Kinase with multiple targets
PRKRA2A	Protein Kinase CAMP-Dependent Type II Regulatory Subunit α	Kinase with multiple targets
SRC	SRC Proto-Oncogene, Non-Receptor Tyrosine Kinase	Kinase with multiple targets
STK39	Serine/Threonine Kinase 39	Kinase with multiple targets
TAB3	TGF-Beta Activated Kinase 1 (MAP3K7) Binding Protein 3	Inflammatory kinase
PIP4P2	Phosphatidylinositol-4,5-Bisphosphate 4-Phosphatase 2	Phosphatase
PPFIBP1	PPFIA Binding Protein 1	Phosphatase interactor
PPME1	Protein phosphatase methylesterase 1 (PME-1)	Phosphatase
PPP1R14A	Protein Phosphatase 1 Regulatory Inhibitor Subunit 14A	Phosphatase
PPP1R2	Protein Phosphatase 1 Regulatory Subunit 2	Phosphatase regulation
PPP1R7	Protein Phosphatase 1 Regulatory Subunit 7	Phosphatase regulation
PPP3CB	Protein Phosphatase 3 Catalytic Subunit Beta	Phosphatase
PPP6R1	Protein Phosphatase 6 Regulatory Subunit 1	Phosphatase regulation

Supplementary Table 9. Abnormally-regulated phosphorylated proteins of the cytoskeleton in phosphoproteome across age groups.

Protein ID	Protein name	Function
ABLIM1	Actin Binding LIM Protein 1	Cytoskeletal component
ADD1	Adducin 1	Cytoskeletal component
ADD2	Adducin 2	Cytoskeletal component
ADGRB1	Adhesion G Protein-Coupled Receptor B1	Synapse component
AHNAK	AHNAK Nucleoprotein	Structural component
ARHGAP32	Rho GTPase Activating Protein 32	Cytoskeletal component
BCAS3	BCAS3 Microtubule Associated Cell Migration Factor	Cytoskeletal component
CAMSAP2	Calmodulin-regulated spectrin-associated protein 2	Cytoskeleton regulator
CCDC6	Coiled-Coil Domain Containing	Cytoskeletal component
CCP110	Centriolar Coiled-Coil Protein 110	Cytoskeleton regulator
CEP170	Centrosomal protein of 170 kDa	Cytoskeletal component
CEP170B	Centrosomal protein 170B	Cytoskeletal component
CFL2	Cofilin 2	Cytoskeleton regulator
CLIP1	CAP-Gly Domain Containing Linker Protein 1	Cytoskeletal component
CLTA	Clathrin Light Chain A	Cytoskeletal component
CRYAB	Crystallin Alpha B	Cytoskeletal component
CTTN	Cortactin	Cytoskeletal regulator
DBN1	Drebrin 1	Cytoskeletal component
DPYSL2	Dihydropyrimidinase Like 2	Cytoskeletal regulator
DPYSL4	Dihydropyrimidinase Like 4	Cytoskeletal regulator
DPYSL5	Dihydropyrimidinase Like 5	Cytoskeletal component
DSP	Desmoplakin	Cytoskeletal component
DVL1	Dishevelled Segment Polarity Protein 1	Cytoskeletal regulator
ERMN	Ermin	Cytoskeletal component
FKBP15	FKBP Prolyl Isomerase Family Member 15	Cytoskeletal regulator
FMNL2	Formin Like 2	Cytoskeletal regulator
FRMD4A	FERM domain-containing protein 4A	Cytoskeletal regulator
FSD1	Fibronectin Type III and SPRY Domain Containing 1	Cytoskeletal regulator
GIT1	GIT ArfGAP 1	Cytoskeletal regulator
HTT	Huntingtin	Cytoskeletal component
ITSN2	Intersectin 2	Cytoskeletal component
JAKMIP3	Janus Kinase and Microtubule Interacting Protein 3	Cytoskeletal component
KANK2	KN Motif and Ankyrin Repeat Domains 2	Cytoskeletal regulator
KIF3A	Kinesin Family Member 3A	Cytoskeletal component
MAP1A	Microtubule-associated protein 1A (MAP-1A)	Cytoskeletal component
MAP1B	Microtubule-associated protein 1B (MAP-1B)	Cytoskeletal component
MAP2	Microtubule-associated protein 2 (MAP-2)	Cytoskeletal component
MAP7D1	MAP7 Domain Containing 1	Cytoskeletal component
MAPRE2	Microtubule Associated Protein RP/EB Family Member 2	Cytoskeletal component
MATR3	Matrin 3	Nuclear matrix protein
MYO18A	Myosin XVIIIA	Cytoskeletal component
NAV1	Neuron Navigator 1	Cytoskeletal component
NDEL1	NudE Neurodevelopment Protein 1 Like 1	Cytoskeletal regulator
NDRG1	N-Myc Downstream Regulated 1	Cytoskeletal component
NEFH	Neurofilament heavy polypeptide (NF-H)	Cytoskeletal component

NEFM	Neurofilament medium polypeptide (NF-M)	Cytoskeletal component
PCLO	Piccolo Presynaptic Cytomatrix Protein	Cytoskeletal component
PCM1	Pericentriolar Material 1	Cytoskeletal component
PHLDB1	Pleckstrin Homology Like Domain Family B Member 1	Cytoskeletal component
PKP2	Plakophilin 2	Cytoskeleton component
PKP4	Plakophilin 4	Cytoskeleton component
PLXNA1	Plexin A1	Cytoskeletal regulator
PPFIA3	PTPRF Interacting Protein Alpha 3	Cytoskeletal component
PPFIBP1	PPFIA Binding Protein 1	Cytoskeletal component
RAB11FIP5	RAB11 Family Interacting Protein 5	Cytoskeletal trafficking
RTKN	Rhotekin	Cytoskeletal regulator
SEPTIN4	Septin 4	Cytoskeletal component
SH2D5	SH2 Domain Containing 5	Membrane protein
SH3KBP1	SH3 Domain Containing Kinase Binding Protein 1	Cytoskeletal regulator
SHANK1	SH3 And Multiple Ankyrin Repeat Domains 1	Cytoskeletal component
SHISA6	Protein shisa-6	Cytoskeletal component
SORBS1	Sorbin And SH3 Domaing Containing 1	Cytoskeletal component
SORBS2	Sorbin And SH3 Domain Containing 2	Cytoskeletal component
SPP1	Osteopontin	ECM component
SRCIN1	SRC kinase-signaling inhibitor 1	Cytoskeletal regulator
SRGAP2	SLIT-ROBO Rho GTPase Activating Protein 2	Cytoskeletal regulator
WIPF2	WAS/WASL-interacting protein family member 2	Cytoskeletal regulator
TIAM1	TIAM Rac1 Associated GEF 1	Cytoskeletal component
TNIK	TRAF2 And NCK Interacting Kinase	Cytoskeletal regulator
TUBA3D	Tubulin Alpha 3D	Cytoskeletal component
TUBB	Tubulin Beta Class I	Cytoskeletal component
TUBB4A	Tubulin Beta 4A Class IVa	Cytoskeletal component
VIM	Vimentin	Cytoskeletal component
WDR47	WD Repeat Domain 47	Cytoskeletal component

Supplementary Table 10. Abnormally-regulated phosphorylation of membrane-associated proteins in phosphoproteome across age groups.

Protein ID	Protein name	Function
AAGAB	Alpha And Gamma Adaptin Binding Protein	Vesicles membranes
ABCA2	ATP Binding Cassette Subfamily A Member 2	Membrane transporter
ABCC8	ATP Binding Cassette Subfamily C Member 8	Membrane transporter
ADRA2A	Adrenoceptor Alpha 2A	Membrane protein
ANK2	Ankyrin-2	Integral membrane protein
ANK3	Ankyrin-3 (Fragment)	Integral membrane protein
ANKRD13D	Ankyrin Repeat Domain 13D	Integral membrane protein
ANKS1B	Ankyrin Repeat and Sterile α Motif Domain Containing 1B	Integral membrane protein
APBA2	Amyloid Beta Precursor Protein Binding Family A Member 2	Vesicular trafficking protein
APLP2	Amyloid Beta Precursor Like Protein 2	Integral membrane protein
ARFGEF2	ADP Ribosylation Factor Guanine Nucleotide Exchange F2	Vesicular trafficking protein
ARGAP1	ADP Ribosylation Factor GTPase Activating Protein 1	Vesicles membranes
ASIC2	Acid Sensing Ion Channel Subunit 2	Membrane transporter
ATP1A1	ATPase Na+/K+ Transporting Subunit Alpha 1	Membrane transporter

ATP2B1	ATPase Plasma Membrane Ca2+ Transporting 1	Membrane transporter
ATP2B2	ATPase Plasma Membrane Ca2+ Transporting 2	Membrane transporter
BASP1	Brain acid soluble protein 1	Membrane component
BCAS1	Brain Enriched Myelin Associated Protein 1	Membrane protein
BYSL	Bystin Like	Membrane cell adhesion
CADPS	Calcium Dependent Secretion Activator	Membrane vesicle protein
CASKIN1	Caskin-1 (CASK-interacting protein 1)	Membrane component
CDH22	Cadherin 22	Membrane cell junction
CLDN11	Claudin 11	Membrane cell junction
CLINT1	Clathrin Interactor 1	Vesicle membrane protein
CNP	2',3'-Cyclic-Nucleotide 3'-Phosphodiesterase	Membrane component
CTNND1	Catenin Delta 1	Membrane cell junction
CTNND2	Catenin Delta 2	Membrane cell junction
DOCK4	Dedicator Of Cytokinesis 4	Membrane cell junction
EHD3	EH domain-containing protein 3	Vesicle membrane protein
EPB41L1	Erythrocyte Membrane Protein Band 4.1 Like 1	Synapse membrane protein
EPB41L3	Erythrocyte membrane protein band 4.1-like 3	Synapse membrane protein
EPN1	Epsin-1 (EPS-15-interacting protein 1)	Vesicles membrane
EXOC2	Exocyst Complex Component 2	Vesicles membrane
FAM234B	Family With Sequence Similarity 234 Member B	Transmembrane protein
FGF12	Fibroblast Growth Factor 12	Membrane protein
FN1	Fibronectin 1	Membrane cell adhesion
GPR34	G Protein-Coupled Receptor 34	Integral membrane protein
GPRIN3	GPRIN Family Member 3	Membrane component
GRIP1	Glutamate Receptor Interacting Protein 1	Membrane component
ITSN1	Intersectin-1 (EH and SH3 domains protein 1)	Membrane/vesicle protein
ITSN2	Intersectin-2 (EH and SH3 domains protein 2)	Membrane/vesicle protein
KCNB1	Potassium voltage-gated channel subfamily B member 1	Membrane ion channel
KCNQ2	Potassium Voltage-Gated Channel Subfamily Q Member 2	Membrane ion channel
KCNQ5	Potassium Voltage-Gated Channel Subfamily Q Member 5	Membrane ion channel
KCTD16	Potassium Channel Tetramerization Domain Containing 16	Membrane ion channel
KIAA1107	AP2-Interacting Clathrin-Endocytosis Protein	Vesicles membrane
KIAA1109	Transmembrane protein KIAA1109	Membrane/vesicle protein
MARCKS	Myristoylated Alanine Rich Protein Kinase C Substrate	Membrane protein
MBP	Myelin Basic Protein	Membrane protein
MFF	Mitochondrial Fission Factor	Vesicles membrane
MTCH1	Mitochondrial Carrier 1	Membrane transporter
NFASC	Neurofascin	Membrane protein
NRCAM	Neuronal Cell Adhesion Molecule	Membrane protein
NUMBL	NUMB Like Endocytic Adaptor Protein	Vesicles membrane
OSBP	Oxysterol Binding Protein	Vesicles transport
PEX5L	Peroxisomal Biogenesis Factor 5 Like	Vesicles membrane
PHF24	PHD Finger Protein 24	Synapse membrane
PLP1	Proteolipid Protein 1	Membrane protein
PPFIA3	PTPRF Interacting Protein Alpha 3	Membrane protein
RAB11A	Member RAS Oncogene Family	Membrane protein
RAB3IP	RAB3A Interacting Protein	Vesicles membrane
RALBP1	RalA Binding Protein 1	Vesicles membrane

RETREG2	Reticulophagy Regulator Family Member 2	Transmembrane protein
RETREG3	Reticulophagy Regulator Family Member 3	Transmembrane protein
RIC8A	RIC8 Guanine Nucleotide Exchange Factor A	Transmembrane protein
RIMS1	Regulating Synaptic Membrane Exocytosis 1	Vesicles membrane
RTN1	Reticulon 1	Membrane trafficking
SCN1A	Sodium Voltage-Gated Channel Alpha Subunit 1	Membrane transporter
SGIP1	SH3-containing GRB2-like protein 3-interacting protein 1	Vesicles membrane
SH3PXD2A	SH3 And PX Domains 2A	Membrane protein
SLC12A5	Solute carrier family 12 member 5	Membrane transporter
SLC2A11	Solute Carrier Family 2 Member 11	Membrane transporter
SLC4A4	Solute Carrier Family 4 Member 4	Membrane transporter
SLC9A6	Solute Carrier Family 9 Member A6	Membrane transporter
SMIM13	Small Integral Membrane Protein 13	Membrane transporter
SNAP91	Synaptosome Associated Protein 91	Vesicles membrane
SNX17	Sorting Nexin 17	Vesicles membrane
SORT1	Sortilin 1	Vesicles membrane
SPTBN1	Spectrin Beta, Non-Erythrocytic 1	Membrane protein
SPTBN2	Spectrin Beta, Non-Erythrocytic 2	Membrane protein
STARD10	StAR Related Lipid Transfer Domain Containing 10	Vesicles membranes
STX1A	Syntaxin 1A	Vesicles membranes
STX1B	Syntaxin 1B	Vesicles membranes
STX7	Syntaxin 7	Vesicles membranes
STXBP1	Syntaxin Binding Protein 1	Vesicles membranes
SV2A	Synaptic Vesicle Glycoprotein 2A	Vesicles membranes
SYN1	Synapsin-1 (Synapsin I)	Membrane component
SYNGR3	Synaptogyrin 3	Vesicles membranes
SYNJ1	Phosphoinositide 5-phosphatase (EC 3.1.3.36)	Vesicles membranes
SYNRG	Synergin Gamma	Vesicles membranes
TBC1D1	TBC1 Domain Family Member 1	Vesicles membranes
TBC1D5	TBC1 Domain Family Member 5	Vesicles membranes
TJP2	Tight junction protein ZO-2	Membrane component
TMCC1	Transmembrane And Coiled-Coil Domain Family 1	Membrane protein
TMEM94	Transmembrane Protein 94	Membrane protein
TNKS1BP1	Tankyrase 1 Binding Protein 1	Membrane protein
TPRG1L	Tumor protein p63-regulated gene 1-like protein	Membrane component
VAPB	VAMP Associated Protein B And C	Vesicles membrane
WASHC2A	WASH Complex Subunit 2A	Vesicles membrane

Supplementary Table 11. Abnormally-regulated phosphorylation of DNA- and RNA-regulating proteins in phosphoproteome across age groups.

Protein ID	Protein name	Function
BCLAF1	BCL2 Associated Transcription Factor 1	Transcription factor
BRD3	Bromodomain Containing 3	DNA/Histone remodeling
DCAF6	DDB1 And CUL4 Associated Factor 6	DNA/nuclear receptors binding
DDX20	DEAD-Box Helicase 20	RNA metabolism
DEK	DEK Proto-Oncogene	DNA metabolism
DGCR8	DGCR8 Microprocessor Complex Subunit	RNA metabolism
EIF3CL	Eukaryotic Translation Initiation Factor 3 Subunit C Like	RNA/protein synthesis

EIF3D	Eukaryotic Translation Initiation Factor 3 Subunit D	RNA/protein synthesis
ESF1	ESF1 Nucleolar Pre-RRNA Processing Protein	RNA metabolism
GPATCH2	G-Patch Domain Containing 2	RNA metabolism
HDAC4	Histone Deacetylase 4	DNA/Histone remodeling
HDAC5	Histone Deacetylase 5	DNA/Histone remodeling
HDLBP	High Density Lipoprotein Binding Protein	DNA/RNA metabolism
HNRNPUL2	Heterogeneous Nuclear Ribonucleoprotein U Like 2	RNA metabolism
LEO1	LEO1 Homolog, Paf1/RNA Polymerase II Complex	RNA metabolism
MLIP	Muscular LMNA Interacting Protein	DNA/RNA metabolism
MRE11	MRE11 Homolog, 2x Strand Break Repair Nuclease	DNA metabolism
NAP1L4	Nucleosome Assembly Protein 1 Like 4	DNA metabolism
NCL	Nucleolin (Protein C23)	RNA/protein synthesis
PCNP	PEST Proteolytic Signal Containing Nuclear Protein	DNA metabolism
PHRF1	PHD And Ring Finger Domains 1	RNA metabolism
PML	PML Nuclear Body Scaffold	Transcription factor
POLR2F	RNA Polymerase II, I And III Subunit F	RNA metabolism
PPIL3	Peptidylprolyl Isomerase Like 3	RNA metabolism
PRPF4B	Pre-MRNA Processing Factor 4B	RNA metabolism
PRR12	Proline-rich protein 12	DNA metabolism
PURA	Purine Rich Element Binding Protein A	DNA metabolism
PWP1	PWP1 Homolog, Endonuclein	DNA/RNA metabolism
R3HDM2	R3H Domain Containing 2	DNA metabolism
RPAP3	RNA Polymerase II Associated Protein 3	RNA metabolism
SCAF1	SR-Related CTD Associated Factor 1	RNA metabolism
SIRT2	Sirtuin 2	DNA metabolism
SLTM	SAFB-like transcription modulator	DNA metabolism
Smarcc2	SWI/SNF complex subunit SMARCC2	DNA metabolism
SPINDOC	Spindlin Interactor and Repressor of Chromatin	DNA metabolism
SRRM1	Serine And Arginine Repetitive Matrix 1	RNA metabolism
SRRM2	Serine/Arginine Repetitive Matrix 2	RNA metabolism
TCEAL3	Transcription elongation factor A protein-like 3	RNA/Protein synthesis
TP53BP1	Tumor Protein P53 Binding Protein 1	DNA metabolism
VIRMA	Vir Like M6A Methyltransferase Associated	RNA metabolism
WDR13	WD Repeat Domain 13	Chromatin structure
XPC	XPC Complex Subunit	DNA metabolism
ZNF704	Zinc finger protein 704	Transcription factor
ZRANB2	Zinc Finger RANBP2-Type Containing 2	RNA metabolism

Supplementary Table 12. Abnormally-regulated phosphorylation of UPS proteins in phosphoproteome across age groups.

Protein ID	Protein name	Function
CAND1	Cullin-associated NEDD8-dissociated protein 1	UPS
FBXO2	F-Box Protein 2	UPS
FBXW9	F-Box and WD Repeat Domain Containing 9	UPS
HECW1	HECT, C2 And WW Domain Containing E3 Ubiquitin Protein Ligase 1	UPS
KBTBD11	Kelch Repeat and BTB Domain Containing 11	UPS
MYCBP2	MYC Binding Protein 2	UPS
Nedd4l	HECT-type E3 ubiquitin transferase (EC 2.3.2.26)	UPS
NEMF	Nuclear Export Mediator Factor	UPS
STT3B	STT3 Oligosaccharyltransferase Complex Catalytic Subunit B	UPS

TRIM33	Tripartite Motif Containing 33	UPS
UBE4B	Ubiquitination Factor E4B	UPS
UBR4	Ubiquitin Protein Ligase E3 Component N-Recognin 4	UPS
USP20	Ubiquitin Specific Peptidase 20	UPS
VCPIP1	Valosin Containing Protein Interacting Protein 1	UPS
ZFAND5	Zinc Finger AN1-Type Containing 5	UPS

Supplementary Table 13. Abnormally-regulated phosphorylation of miscellaneous proteins in phosphoproteome across age groups.

Protein ID	Protein name	Function
ACACA	Acetyl-CoA carboxylase 1 (ACC1)	Fatty acid biosynthesis
ATG2B	Autophagy-related protein 2 homolog B	Autophagy
ARGLU1	Arginine And Glutamate Rich 1	Nuclear receptor regulator
CBR1	Carbonyl Reductase 1	Multitarget reductase
CBR3	Carbonyl Reductase 3	Multitarget reductase
CCDC177	Coiled-Coil Domain Containing 177	Chromosome structure
CCDC92	Coiled-Coil Domain Containing 92	Antiviral protein
FAM131B	Family With Sequence Similarity 131 Member B	MAPK signaling
FDX1	Ferredoxin 1	Electron transfer activity
HMGCS1	3-Hydroxy-3-Methylglutaryl-CoA Synthase 1	Lipid metabolism
IGFBP5	Insulin Like Growth Factor Binding Protein 5	Growth factor
LIPE	Lipase E, Hormone Sensitive Type	Lipid metabolism
LRRFIP2	LRR Binding FLII Interacting Protein 2	Inflammation
LUZP1	Leucine Zipper Protein 1	Nuclear unknown function
MCF2L	MCF.2 Cell Line Derived Transforming Sequence Like	GTPase modulator
MCRIP1	MAPK Regulated Corepressor Interacting Protein 1	MAP kinase signaling
NGEF	Neuronal Guanine Nucleotide Exchange Factor	GTPase activator
PDCD5	Programmed Cell Death 5	Apoptosis
PDE4B	Phosphodiesterase 4B	cAMP signaling pathway
PLCB1	Phospholipase C Beta 1	PI Metabolism
PLEKHA6	Pleckstrin Homology Domain Containing A6	PI Metabolism
PTGES3	Prostaglandin E Synthase 3	Chaperone
RABL6	RAB, Member RAS Oncogene Family Like 6	GTPases
RALGAPA1	Ral GTPase Activating Protein Catalytic Subunit Alpha 1	GTPase regulator
RANBP1	RAN Binding Protein 1	Nuclear transport
RAP1GAP2	RAP1 GTPase Activating Protein 2	GTPase regulator
RASGRF1	Ras Protein Specific Guanine Nucleotide Releasing Factor 1	GTPase regulator
SGSM1	Small G Protein Signaling Modulator 1	GTPase regulator
SGTA	Small Glutamine Rich Tetratricopeptide Repeat Co-Chaperone α	Unknown function
SHC3	SHC Adaptor Protein 3	Trk signaling
SRGAP3	SLIT-ROBO Rho GTPase Activating Protein 3	GTPase activator
ST13	ST13 Hsp70 Interacting Protein	Heat shock protein modulator
SUDS3	SDS3 Homolog, SIN3A Corepressor Complex Component	Nuclear component regulator
TTC7B	Tetratricopeptide Repeat Domain 7B	Signaling pathway
YWHAB	Tryptophan 5-Monooxygenase Activation Protein Beta	Monooxygenase activity
YWHAG	Tryptophan 5-Monooxygenase Activation Protein Gamma	Monooxygenase activity
YWHAZ	Tryptophan 5-Monooxygenase Activation Protein Zeta	Monooxygenase activity
